# Effects of Probiotics at the Interface of Metabolism and Immunity to Prevent Colorectal Cancer-Associated Gut Inflammation: A Systematic Network and Meta-Analysis With Molecular Docking Studies

**DOI:** 10.3389/fmicb.2022.878297

**Published:** 2022-05-27

**Authors:** Sinjini Patra, Nilanjan Sahu, Shivam Saxena, Biswaranjan Pradhan, Saroj Kumar Nayak, Anasuya Roychowdhury

**Affiliations:** ^1^Biochemistry and Cell Biology Laboratory, School of Basic Sciences, Indian Institute of Technology Bhubaneswar, Odisha, India; ^2^National Institute of Science Education and Research (NISER) Bhubaneswar, Homi Bhabha National Institute (HBNI), Odisha, India; ^3^S. K. Dash Center of Excellence of Biosciences and Engineering & Technology (SKBET), Indian Institute of Technology Bhubaneswar, Odisha, India

**Keywords:** gut microbiome, colorectal cancer, probiotic intervention, bacteriocins, meta-analysis, gene network analysis, systematic review, molecular docking

## Abstract

**Background:**

Dysbiosis/imbalance in the gut microbial composition triggers chronic inflammation and promotes colorectal cancer (CRC). Modulation of the gut microbiome by the administration of probiotics is a promising strategy to reduce carcinogenic inflammation. However, the mechanism remains unclear.

**Methods:**

In this study, we presented a systematic network, meta-analysis, and molecular docking studies to determine the plausible mechanism of probiotic intervention in diminishing CRC-causing inflammations.

**Results:**

We selected 77 clinical, preclinical, *in vitro*, and *in vivo* articles (PRISMA guidelines) and identified 36 probiotics and 135 training genes connected to patients with CRC with probiotic application. The meta-analysis rationalizes the application of probiotics in the prevention and treatment of CRC. An association network is generated with 540 nodes and 1,423 edges. MCODE cluster analysis identifies 43 densely interconnected modules from the network. Gene ontology (GO) and pathway enrichment analysis of the top scoring and functionally significant modules reveal stress-induced metabolic pathways (JNK, MAPK), immunomodulatory pathways, intrinsic apoptotic pathways, and autophagy as contributors for CRC where probiotics could offer major benefits. Based on the enrichment analyses, 23 CRC-associated proteins and 7 probiotic-derived bacteriocins were selected for molecular docking studies. Results indicate that the key CRC-associated proteins (e.g., COX-2, CASP9, PI3K, and IL18R) significantly interact with the probiotic-derived bacteriocins (e.g., plantaricin JLA-9, lactococcin A, and lactococcin mmfii). Finally, a model for probiotic intervention to reduce CRC-associated inflammation has been proposed.

**Conclusion:**

Probiotics and/or probiotic-derived bacteriocins could directly interact with CRC-promoting COX2. They could modulate inflammatory NLRP3 and NFkB pathways to reduce CRC-associated inflammation. Probiotics could also activate autophagy and apoptosis by regulating PI3K/AKT and caspase pathways in CRC. In summary, the potential mechanisms of probiotic-mediated CRC prevention include multiple signaling cascades, yet pathways related to metabolism and immunity are the crucial ones.

## Graphical Abstract

Probiotic intervention to mitigate colorectal cancer. 
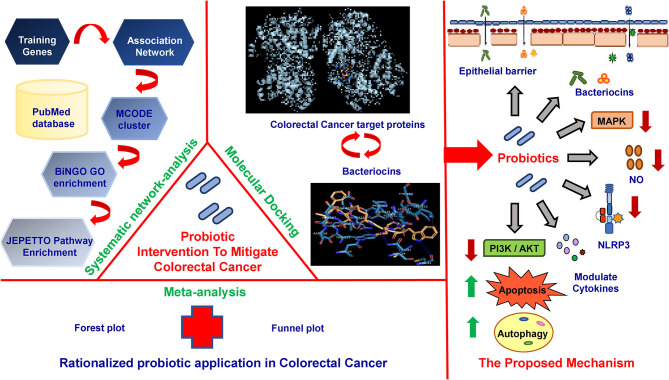

## Introduction

Colorectal cancer (CRC) has emerged as the most common malignancy of the gastrointestinal (GI) tract (Rawla et al., 2019). The disease is diagnosed at a rate of about 10% annually and ranks second as the major cancer-causing death worldwide (Dekker et al., 2019; Sung et al., 2021). According to the World Health Organization (WHO), a significantly increased death rate with about 75 million people affected by CRC would be detected by 2030 (Kuipers et al., 2015). Such a threatening epidemiology of CRC strongly suggests an urgent investigation into the novel strategy for CRC prevention and treatment.

The interplay of genetic and environmental risk factors (lifestyle and food habits) fosters CRC (Yang et al., 2019). Hereditary factors are responsible for around 10–20% of all patients with CRC, suggesting the sporadic nature of the disease (Chen et al., 2018). Recent reports indicate that specific gut bacteria, or imbalances in gut bacterial populations (dysbiosis), could contribute to the development of CRC (Saus et al., 2019). The commensal intestinal microflora enriches host immunity metabolism and serves as a protective barrier against pathogens (Jandhyala et al., 2015). The gut microorganisms release beneficial metabolites such as short-chain fatty acids (SCFAs) and bacteriocins with numerous health-promoting properties. The SCFAs are the preferred energy source of colonocytes and maintain immune homeostasis, whereas bacteriocins are antimicrobial peptides that prevent the growth of inflammation-associated pathogenic bacteria (Louis et al., 2014; O'keefe, 2016; Khan et al., 2020). However, an unhealthy diet, broad-spectrum antibiotics, and lifestyle behaviors (less exercise, smoking, consumption of alcohol, and progressive aging of the population) often cause dysbiosis and damage the protective gut barrier. This allows the pathogens and their carcinogenic metabolites to enter and activate the host immune system, commencing continuous inflammatory processes that lead to the development of CRC (Conlon and Bird, 2014; Wong and Yu, 2019). A plethora of clinical studies report on the imbalanced gut microbiota of patients with CRC compared to the normal ones (Cruz et al., 2020).

Interestingly, the consumption of beneficial bacteria or probiotics with food supplements can reconstitute the disrupted equilibrium of the gut microbiota (George Kerry et al., 2018). Clinically, it is also suggested that probiotic formulations are useful in attenuating CRC risk and postoperative complications in patients with CRC (Kahouli et al., 2013; Shokryazdan et al., 2014). Although probiotics are reported to downregulate CRC-causing chronic inflammatory and carcinogenic activities in the colon with a decreased recurrence of treatment toxicity, the detailed mechanism is obscure.

Computational models and network-based methodologies can generate association networks using data from the published literature and databases to find new information from complex biological interactions. In this way, one can recognize the hidden link, visualize the global picture of the relevant biological interactions, and identify the major target molecules involved in such interactions. Furthermore, molecular docking studies can assist in the estimation of the ligand-target associations at the molecular level (Pinzi and Rastelli, 2019).

In this study, we identified 36 probiotic bacteria and 135 training genes by analyzing 77 clinical, preclinical, *in vitro*, and *in vivo* reports on anti-CRC probiotic treatment using a data-mining approach. The meta-analysis study rationalizes the use of probiotics in the prevention and reduction of postoperative infections in patients with CRC. An association network with 540 nodes and 1,423 edges on probiotics CRC-related candidate genes is generated by the text-mining approach. MCODE derives 43 closely interconnected modules from the network. Functional annotation, GO, and pathway enrichment analysis of the 6 highest scoring modules reveal that stress-induced metabolic pathways (JNK and MAPK), immunomodulatory pathways, intrinsic apoptotic pathway, and autophagy are the principal domains where probiotic intervention could be significantly effective for CRC pathogenesis. Further molecular docking studies decipher the interactions between the key target proteins of CRC and probiotic-derived bacteriocins and reaffirm our network analysis. Strikingly, bacteriocins, namely, plantaricin JLA-9, plantaricin W, lactococcin A, and lactococcin mmfii, show the highest binding affinity with the crucial CRC promoting protein COX-2, initiator caspase CASP9 of the apoptosis pathway, stress-induced metabolic pathway mediator PI3K, and immunomodulatory IL18R. Finally, based on our analysis, we propose a mechanistic model for the probiotic-mediated reduction of carcinogenic gut inflammation. Thus, the study provides a comprehensive mechanistic insight into the role of probiotics to combat CRC and endorses the rationale for probiotic application as an alternative treatment and preventive strategy for the disease.

## Materials and Methods

**Figure 1:**
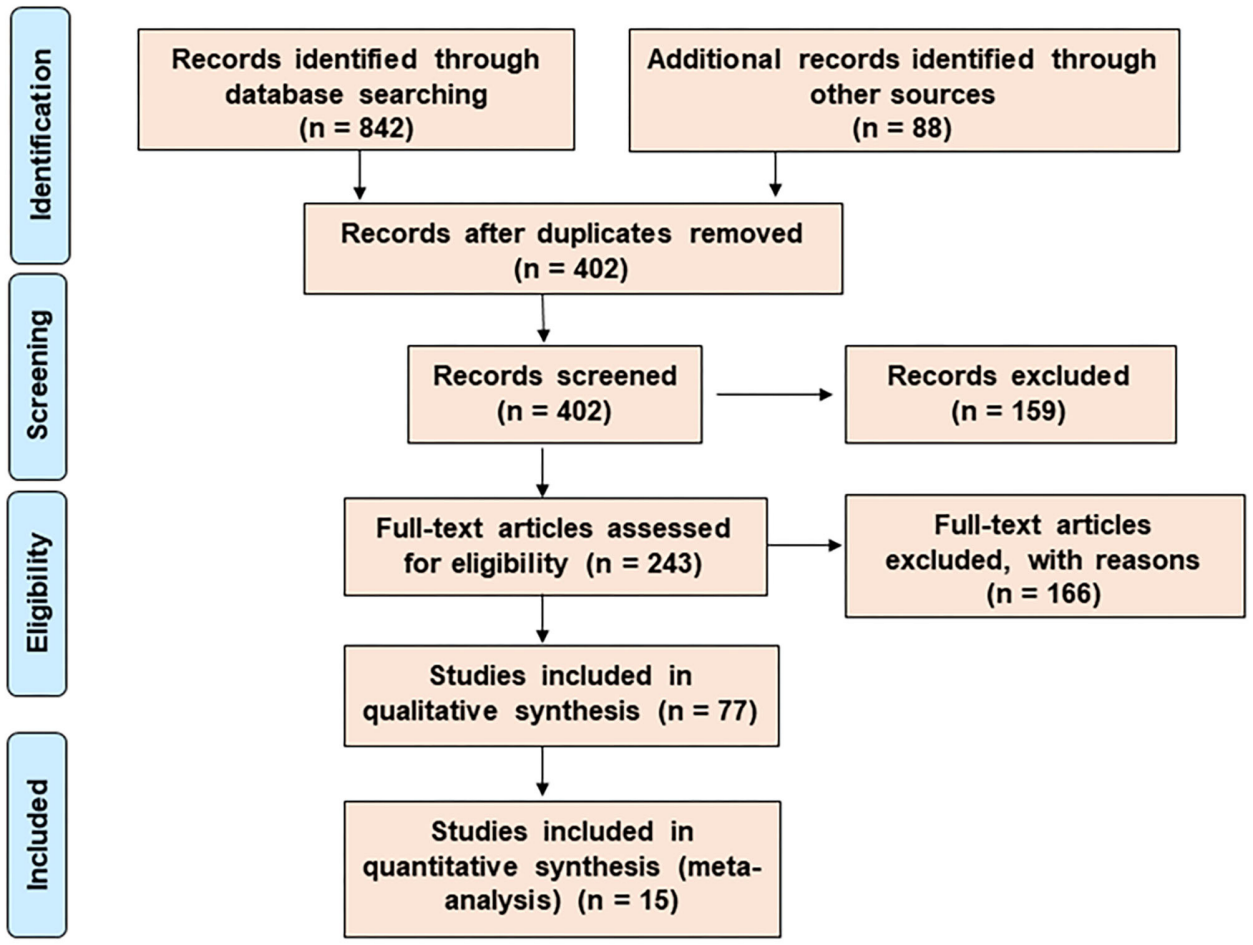
The search and selection process for the systematic review. The PRISMA diagram represents the method for literature search and selection.

A meta-analysis and a systematic network were considered to assess the bioprotective mechanism of probiotics against CRC. Published data were analyzed through data- and text-mining–based approaches. The data-mining strategy was used for automatic detection of disease patterns from structured databases and finding out the functional genes that were comparatively unexplored. However, for more efficient screening, text-mining was further used. This novel information retrieval resource simultaneously constructs the interaction network from unstructured biomedical texts. Since the sole use of text-mining strategy might introduce biases toward dynamically studied disease phenotypes, a unique combination of data- and text-mining techniques was implemented to include the less explored studies. The Preferred Reporting Items for Systematic Reviews and Meta-Analyses (PRISMA) guideline was followed for the literature survey of this study (Figure 1). Supplementary Figure 1 summarizes the entire methodology.

### Meta-Analysis Establishes the Rationale for Probiotic Intervention as an Alternative Strategy for the Prevention and Alleviation of CRC-Related Inflammation

**Figure 2:**
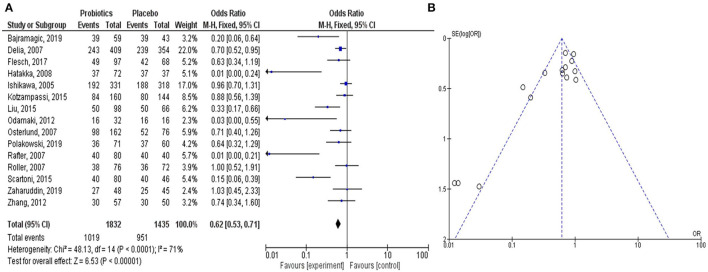
Meta-analysis study justifies the efficacy of the probiotic intervention in the treatment and prevention of CRC-promoting gut inflammation. The Forest plot shows 95% confidence intervals and pooled mean difference to evaluate the result of placebo controls vs. probiotics-based treatment (after heterogeneity adjustment) on patients with CRC **(A)**. The Funnel plot of the effect size plotted with the standard error examines publication bias to show the effect of probiotics on CRC in various clinical trials **(B)**. The pooled effect size is represented by the perpendicular line to the x-axis. Positive or negative bias is represented by the studies outside the triangle. Thus, the substantial asymmetry in the funnel plot signifies the absence of publication bias.

The meta-analysis study rationalized the therapeutic contribution of probiotics in the alleviation of colorectal carcinogenesis (Figure 2). In the study, clinical trials on probiotic application in patients with CRC were analyzed from the literature. The forest plot represented the heterogeneity in pooled estimates of the individual study outcomes. Begg's funnel plot was implemented to evaluate the publication bias. The detailed method of the meta-analysis study is described in the Supplementary Material.

### Data Sources and Search Strategy Using the Data-Mining Approach

The data-mining evaluated the studies conducted on colorectal carcinogenesis associated with gut inflammation and the administration of specific probiotic strains to ameliorate the disease severity. Data-mining or manual analysis was performed on the published articles listed in the PubMed archive (http://www.ncbi.nlm.nih.gov/pubmed/) using EndNote X7 (Bld 7072) (endnote.com), a reference management software. More details of the search process and keywords are described in the Supplementary Materials. The training genes, which have already been established to be associated with CRC-causing gut inflammation and probiotic strains, were selected for the text-mining study.

### Selection of the Genes and Network Construction Using the Text-Mining Approach

To reaffirm that the training genes were involved in the probiotics-mediated alleviation of carcinogenic gut inflammation, the text-mining approach was carried out using Cytoscape 3.7.1 App and Agilent Literature Search 3.1.1 beta (LitSearch version 2.69). This could retrieve the functionally related genes from the existing literature in PubMed and construct the network as described in the Supplementary Material.

### Data Extraction Using Molecular Complex Detection Analysis

The Molecular Complex Detection (MCODE) plug-in of Cytoscape was applied to identify firmly associated nodes from a very closely connected network, which helped in recognizing biological modules (Bader and Hogue, 2003). The method is provided in the Supplementary Material. MCODE modules with a score >4 and nodes ≥4 were considered for further functional annotation analysis.

### Quantitative Data Synthesis Using NetworkAnalyzer

NetworkAnalyzer tool in Cytoscape procured details of the topology parameter for the association network and MCODE modules according to the procedure in the Supplementary Material. Next, we used GeneCards (version 4.14) to determine gene-centric data of the annotated and predicted human genes, which provided the pathways and functions related to the candidate genes.

### Assessment of Gene Ontology Overrepresentation: The Biological Networks Gene Ontology (BiNGO) Analysis

Gene ontology functional enrichment analysis was used to identify enriched biological processes associated with colorectal carcinogenesis-associated gut inflammation using BiNGO (version 3.0.3) in Cytoscape 2.8.0 (http://www.cytoscape.org/) using the method depicted in the Supplementary Material.

### Identification of Significant Candidate Gene-Associated Enriched Pathways Involved With CRC-Associated Inflammation

The pathways contributing to gut inflammation leading to CRC development were determined using the Java Enrichment of Pathways Extended to Topology (JEPETTO) tool, a Cytoscape 3.x plug-in as described in the Supplementary Materials.

### Molecular Docking Analysis

Molecular docking was carried out to determine the direct interaction of selected probiotic secretory molecules (bacteriocins) with key CRC-associated proteins. The molecular docking method we applied in this study was used to replicate a few protein-ligand binding affinity scores of the previously published computational studies to get confidence in the method (Anwar et al., 2020; Sentürk et al., 2020). The 3D structures of the ligands (probiotic-derived bacteriocins) in “.sdf” format were obtained from PubChem (https://pubchem.ncbi.nlm.nih.gov/). The structural optimization of the ligands was done by energy minimization using Open Babel (version V3.3.1) and the MM2 force field. CRC-associated key protein structures were obtained in “.pdb” format from the Protein Data Bank (PDB, https://www.rcsb.org/). Molecular docking studies were performed in AutoDock Vina (version V1.1.2). The unique features of AutoDock Vina (version V1.1.2) only accept PDBQT format [Partial Charge (Q) & Atom Type (T)] as input for docking. The MGL tool (version V1.5.4) in AutoDock Vina was used to convert the structures from PDB to PDBQT. The entire target protein was considered the search space in the simulation of ligand binding to the protein. The final mean affinity score for each binding process was obtained by repeating the simulation of each ligand 100 times. After that, the best affinity score for protein–ligand interaction of each ligand was selected to visualize the site of binding of the ligands with the proteins in the PyMOL (https://pymol.org/).

## Results

### Meta-Analysis Established the Efficacy of Probiotic Application to Attenuate CRC-Associated Gut Inflammation

In all the clinical trials, the severity and duration of CRC-causing inflammation were compared between individual placebo-controlled groups and probiotic or synbiotic treatment to show the probiotic effects in reducing the carcinogenic inflammation (Table 1) (Ishikawa et al., 2005; Delia et al., 2007; Osterlund et al., 2007; Rafter et al., 2007; Roller et al., 2007; Hatakka et al., 2008; Odamaki et al., 2012; Zhang et al., 2012; Kotzampassi et al., 2015; Liu and Huang, 2015; Scartoni et al., 2015; Flesch et al., 2017; Bajramagic et al., 2019; Polakowski et al., 2019; Zaharuddin et al., 2019). According to the forest plot, calculated *I*^2^ value was 71% and *Q* (chi^2^) statistics was 48.13 (*p* < 0.0001) (Figure 2A). As indicated by the *I*^2^ value, the observed statistical heterogeneity could be due to diverse intervention periods of the probiotics, patient's age and sex, and variation in the disease conditions in clinical trials. The overall effect size (*Z*) of the analysis was 6.53, with *p* < 0.00001. The confidence level (95% CI), weight percentage, and effect size (ES) of individual clinical trials were also shown. Thus, in our meta-analysis study, the forest plot statistically signified the effects of probiotic treatment on gut inflammation that triggers CRC. The funnel plot of the meta-analysis study (Figure 2B) indicated the minimal publication bias in all the selected randomized, double-blind, placebo-controlled human clinical trials on probiotic treatment for patients with CRC. The funnel plot with probiotic application on patients with CRC was symmetrical, demonstrating the least asymmetry in the analysis.

**Table 1:** Probiotics or probiotic formulations, applied in prevention and treatment of colorectal cancer (CRC) in human clinical trials.

**References**	**Probiotic strain**	**Formulation**	**Age group**	**Dosage**	**Mode of administration**	**Function**
Bajramagic et al. (2019)	*Lactobacillus acidophilus*	*L. acidophilus, L. casei, L. plantarum, L. rhamnosus, B. lactis, B. bifidum, B. breve, S. thermophilus*	Adults (patients with colorectal adenocarcinoma)	2 ×1 capsules from the third post-operative day during the next thirty days, and then 1 ×1 for two weeks each next month to a total of one year	Oral	Significantly reduced post-operative complications in the localization of tumors on the rectum and the ascending colon
Delia et al. (2007)	*Lactobacillus acidophilus*	*L. casei, L. plantarum, L. acidophilus, L. delbruekii* subsp. *bulgaricus, B. longum, B. breve, B. infantis*, and *S. salivarius* subsp. *thermophilus*	Adults (Cancer patients)	1 sachet of VSL#3 with 450 billion/g of viable lyophilized bacteria starting from the first day of radiation therapy until the end of the scheduled cycles of radiation therapy	Oral	Protected cancer patients against the risk of radiation-induced diarrhea
Flesch et al. (2017)	*Lactobacillus acidophilus*	*L. acidophilus* NCFM*, L. rhamnosus* HN001*, L. paracasei* LPC-37*, B. lactis* HN019 and fructooligosaccharides (FOS)	Adults 60 −65 years (patients with colorectal cancer)	2 sachets each with 1 ×10*^9^* cfu *L. acidophilus* NCFM, 1 ×10*^9^* cfu *L. rhamnosus* HN001, 1 ×10*^9^* cfu *L. paracasei* LPC-37, 1 ×10*^9^* cfu *B. lactis* HN019 and 6 g fructo-oligosaccharide (FOS), twice a day for 5 days before and 14 days after surgical procedure	Oral	Significantly reduced post-operative infection rates in patients with colorectal cancer
Hatakka et al. (2008)	*Lactobacillus rhamnosus GG*	*L. rhamnosus* (LC705) DSM7061, *Propionibacterium freudenreichii* ssp. *shermanii* JS (PJS), DSM7067	Adults 24–55 years (healthy)	1 capsule containing viable 2 ×10^10^ cfu of each strain LC705 and PJS daily for 4 weeks	Oral	Increased the fecal counts of *lactobacilli* and *propionibacteria* and decreased the activity of β-glucosidase with increasing counts of *propionibacteria*
Ishikawa et al. (2005)	*Lactobacillus casei*	*L. casei* strain Shirota	Adults 40–65 years (cancer patients)	1 g powder with 1 ×10^10^ cfu viable cells after every meal for 4 years after removal of colorectal tumor	Oral	Suppressed the development of colorectal tumors
Kotzampassi et al. (2015)	*Lactobacillus acidophilus*	*L. acidophilus* LA-5, *L. plantarum, B. lactis* BB-12 and *S. boulardii* (LactoLevure UniPharma, Athens, Greece)	Adults ≥18 years (patients diagnosed with colorectal cancer programmed for open surgery for colorectal cancer)	1 capsule with 1.75 ×10^9^ cfu *L. acidophilus* LA-5, 0.5 ×10^9^ cfu *L. plantarum*, 1.75 ×10^9^ cfu *B. lactis* BB-12, 1.5 ×10^9^ cfu *S. boulardii*, twice a day with 100 ml of drinking water on the day of operation and for the next 14 consecutive days	Oral	Significantly decreased the risk of post-operative complications, like mechanical ventilation, infections, and anastomotic leakage in colorectal cancer patients
Liu and Huang (2015)	*Bifidobacterium tetragenous*	*B. tetragenous viable bacteria*	Adults 50–70 years (cancer patients)	4 B. tetragenous viable bacteria tablets (Siliankang®), 3 times per day and continued for 4 weeks as one cycle	Oral	Effective for treating cancer patients with functional constipation, and is safe and well-tolerated
Odamaki et al. (2012)	*Bifidobacterium longum*	*B. longum BB536, L. lactis, S. thermophilus* and *L. delbrueckii* subsp. *bulgaricus*	Adults 30–50 years (healthy)	Yogurt with 1.0 ×10^9^ cfu lactic acid bacteria, 4.27 ×10^8^, and more than 1.25 ×10^8^ cfu living *B. longum* BB536 daily for 8 weeks	Oral	Eliminated enterotoxigenic Bacteroides fragilis (ETBF) strains in the microbiota, associated with acute and persistent diarrheal disease, inflammatory bowel disease, and colorectal cancer
Osterlund et al. (2007)	*Lactobacillus rhamnosus GG*	*L. rhamnosus GG* (ATCC 53103, Gefiluss, Valio Ltd, Helsinki, Finland)	Adults 31–75 years (cancer patients)	1 gelatine capsule with 1–2 ×10^10^ cfu *L. rhamnosus GG* swallowed or dissolved in cold milk or juice twice daily during the 24 weeks of adjuvant cancer chemotherapy	Oral	Reduced the frequency of severe 5-FU-based chemotherapy-related diarrhea
Polakowski et al. (2019)	*Lactobacillus acidophilus*	*L. acidophilus* NCFM*, L. rhamnosus* HN001*, L. casei* LPC-37, *B. lactis* HN019, and fructo-oligosaccharides	Adults 51–68 years (cancer patients)	100 ml of water with Simbiflora containing 6 g of fructo-oligosaccharide and 1 ×10^9^ cfu each of *L. acidophilus* NCFM, *L. rhamnosus* HN001, *L. casei* LPC-37, and *B. lactis* HN019, twice a day for 7 days prior to surgical procedure	Oral	Reduced levels of pro-inflammatory C-reactive protein, IL6, antibiotic use and length of hospital stay after surgery, and morbidity in colorectal cancer patients in the post-operative period.
Rafter et al. (2007)	*Lactobacillus rhamnosus GG*	*L. delbreuckii* subsp. rhamnosus strain GG (LGG), *B. lactis* Bb12 (BB12), and oligofructose enriched inulin (SYN1)	Adults 46–68 years (cancer patients)	1 ×10^10^ cfu/g product daily for 12 weeks after resection of colon cancer	Oral	Increased *Bifidobacterium, Lactobacillus*, secretion of IL-2 by PBMC, and production of IFN-γ. Decreased *Clostridium perfringens*, significantly reduced colorectal proliferation and induced necrosis in colonic cells, improved epithelial barrier function in patients in cancer patients.
Roller et al. (2007)	*Lactobacillus rhamnosus GG*	*L. rhamnosus GG* (LGG), *B. lactis Bb12* (BB-12), and inulin enriched with oligofructose	Adults 54–68 years (cancer patients)	1 sachet with 1 ×10^10^ cfu *L. rhamnosus GG* (LGG), 1 ×10^10^ cfu *B. lactis* Bb12 (BB-12), and 10 g inulin enriched with oligofructose, daily for 12 weeks after resection of colon cancer	Oral	Prevented increase in IL-2 secretion by activated PBMC, increased capacity of PBMC to produce IFN-γ
Scartoni et al. (2015)	*Lactobacillus acidophilus*	*L. casei, L. acidophilus*, galacto-oligosaccharides, zinc, vitamin B1, vitamin B2, vitamin B6, and nicotinamide	Adults >18 years candidates to receive radiation therapy (radical or neo/adjuvant) for colorectal, cervical, anal, endometrial, and prostate cancer	1 ×10 ml vial, Dixentil (Gamfarma srl, Milano Italy) with 500 mg of galacto-oligosaccharides, 10 mg *L. casei*, 10 mg *L. acidophilus*,10 mg zinc, 1 mg vitamin B1, 1 mg vitamin B2, 1 mg vitamin B6, and 10 mg nicotinamide, daily during radiotherapy treatment	Oral	Prevented and reduced radiation-related or radiation-induced gastrointestinal disorders in colorectal cancer patients
Zaharuddin et al. (2019)	*Bifidbacterium bifidum*	*L. acidophilus* BCMC® 12,130, *L. lactis* BCMC® 12,451, *L. casei* subsp. BCMC® 12,313, *B. longum* BCMC® 02120, *B. bifidum* BCMC® 02290 and *B. infantis* BCMC® 02129	Adults ≥18 years (patients diagnosed with colorectal cancer)	107 mg of probiotics with 30 ×10^9^ cfu of six viable *Lactobacillus* and *Bifidobacteria* strains, 4 weeks after surgery twice daily for 6 months	Oral	Reduced systemic production of pro inflammatory cytokines, TNF-α, IL-17A, IL-17C, IL-22, IL-10, and IL-12 and prevented post-surgical complications in colorectal cancer patients
Zhang et al. (2012)	*Bifidobacterium longum*	*B. longum, L. acidophilus* and *Enterococcus faecalis* (Shanghai Sine Wangxiang Pharmaceutical Co. (Shanghai, China)	Adult 45–90 years (patients diagnosed with colorectal adenocarcinoma and elective radical CRC resection with laparotomy)	Bifid triple viable capsule, each with 0.21 g (10^8^ cfu/g) *B. longum, L. acidophilus*, and *Enterococcus faecalis*, 3 times a day for 3 days before radical colorectal resection	Oral	Maintained the intestinal flora, restricted bacterial translocation from the intestine minimized the post-operative occurrence of infectious complications, enhanced systemic/localized immunity and attenuated systemic stress response

### Identification of Probiotic Bacteria and the Respective Training Genes Associated With Oncogenic Gut Inflammation

The results of the literature search were summarized by the PRISMA diagram (Figure 1). The efficient exploratory technique like data-mining found 402 (after the removal of duplicate records) articles with clinical, preclinical, *in vitro*, and *in vivo* data associated with CRC-promoting gut inflammation and probiotic treatment (Femia et al., 2002; Lan et al., 2007; Roller et al., 2007; Mahkonen et al., 2008; Chen et al., 2012; Daniluk et al., 2012; Dong et al., 2012; Talero et al., 2015; An and Ha, 2016; Irecta-Nájera et al., 2017; Madempudi and Kalle, 2017; Sharifi et al., 2017; Deol et al., 2018; Kaeid Sharaf and Shukla, 2018; Oh et al., 2018, 2020; Walia et al., 2018; Agah et al., 2019; An et al., 2019; Huang et al., 2019; Karimi Ardestani et al., 2019; Cruz et al., 2020; Parisa et al., 2020; Ewaschuk et al., 2006; Ko et al., 2007; Chen et al., 2009, 2015, 2017, 2020; Baldwin^*^ et al., 2010; Bertkova et al., 2010; Ma et al., 2010; Borowicki et al., 2011; Foo et al., 2011; Bassaganya-Riera et al., 2012; Liu et al., 2013; Walia et al., 2015; Cousin et al., 2016; Del Carmen et al., 2016; Do et al., 2016; Gamallat et al., 2016; Konishi et al., 2016; Kuugbee et al., 2016; Tiptiri-Kourpeti et al., 2016; Chung et al., 2017, 2019; Djaldetti and Bessler, 2017; Mi et al., 2017; Chondrou et al., 2018; Golkhalkhali et al., 2018; Mendes et al., 2018; Norouzi et al., 2018; Song et al., 2018; Fahmy et al., 2019; Hadad et al., 2019; Heydari et al., 2019; Rong et al., 2019; Saito et al., 2019; Dong et al., 2020; Kim et al., 2020; Lee et al., 2020; Yue et al., 2020). The use of particular MeSH terms in EndNote X7 excluded all non-specific search results, ensuring the accuracy of the mining study. Our analysis shortlisted around 36 probiotic bacteria that have been shown to reduce gut inflammation and 135 training genes of interest involved in carcinogenic gut inflammation (Table 2). These genes were used for further investigation and network studies.

**Table 2:** Identified probiotic strains and the corresponding training gene sets associated with CRC-triggering gut inflammation.

**References**	**Probiotic strain**	**Training genes**
Cruz et al. (2020)	*S. thermophilus* BT01, *B. breve* BB02, *B. animalis* subsp. *lactis* BL03, *B. animalis* subsp. *lactis* BI04, *L. acidophilus* BA05, *L. plantarum* BP06, *L. paracasei* BP07, *L. helveticus* BD08	*il-2, il-4, il-6, il-10, il-17*, *tnf, ifn*
Roller et al. (2007)	*L. rhamnosus* GG (LGG), *B. lactis* Bb12 (BB-12)	*il-2/ifn-g, tnf-α/il-12, il-10, pge2, tgf-β1*
Kaeid Sharaf and Shukla (2018)	*L. acidophilus* NCDC #15, *L. rhamnosus* GG MTCC #1408	*ctnnb1, nf-κb1, cox-2*
Mahkonen et al. (2008)	*L. acidophilus* 74-2, *L. rhamnosus* GG, *B. lactis* sp. 420 (Bif420)	*cox-1, cox-2*
Deol et al. (2018)	*L. acidophilus* MTCC 5401	*cox-2, inos, c-myc*
Oh et al. (2018), Dong et al. (2012)	*L. rhamnosus* 4B15 (4B15), *L. acidophilus, B. bifidum* MF 20/5, *L. gasseri* 4M13 (4M13), *L. casei* Shirota, *L. rhamnosus* GG, *L. plantarum* NCIMB 8826, *L. reuteri* NCIMB 11951, *B. longum* SP 07/3	*il-6, il-1β, il-10, il-4, il-13, tnf, tgfβ1*
Walia et al. (2018)	*L. plantarum* (AdF10), *L. rhamnosus* GG (LGG)	*gsh, gpx, gst, sod1, cat, tp53, p21, bcl2, bax, casp3, casp9*
Sharifi et al. (2017)	*L. paracasei, L. kefiri, L. parabuchneri, Acetobacter lovaniensis*	*tgfβ1, tnf-α, bcl2, bax, p53, p21*
Madempudi and Kalle (2017)	*B. coagulans Unique* IS2	*bcl2, bax, tp53, parp1, cytc, actb, casp-3*
Irecta-Nájera et al. (2017)	*E. durans, L. casei* ATCC 393	*odc1*
Huang et al. (2019)	*S. thermophilus, L. rhamnosus, L. acidophilus, L. casei, B. bifidum, B. longum, L. casei* variety *rhamnosus* Lcr35, *L. acidophilus, B. bifidum* LaBi	*tnf-α, il-1β, ifnγ, il-6, il-10, il-1, il-17*
Daniluk et al. (2012)	*L. helveticus, S. thermophilus, L. bulgaricus*	*bcl2, bax, tlr2, tlr4, nod1, nod2, nlrp6*
Karimi Ardestani et al. (2019)	*L. paracasei* IBRC M10784, *L. brevis* IBRC M1079	*bcl2, bax, casp-3, casp-9, b-actin*
Talero et al. (2015)	*S. thermophilus* DSM24731, *L. acidophilus* DSM24735, *L. delbrueckii* spp. *bulgaricus* DSM24734, *L. paracasei* DSM24733, *L. plantarum* DSM24730, *B. longum* DSM24736, *B. infantis* DSM24737, *B. breve* DSM24732	*tnf-α, il-1b, il-6, il-10, cox-2*
Chen et al. (2012)	*L. acidophilus* NCFM (La)	*cxcr4, cxcl12, mhci*
Lan et al. (2007)	*Propionibacterium freudenreichii* subsp. *freudenreichii* strain TL142	*casp-8, casp-9, casp-3, casp-7, rip1, parp, cyt c, bax, vdac*
Femia et al. (2002)	*B. lactis* (Bb12), *L. rhamnosus* (LGG)	*gst, cox-2, inos*
Parisa et al. (2020)	*B. breve, L. reuteri, L. plantarum, L. rhamnosus, L. brevis, B. bifidum, B. longum*	*egfr, her-2, ptgs-2, cox-2*
Oh et al. (2020)	*L. gasseri* 505	*sod, cat, gr, gpx, tnf-α, cox-2, mpo, nf-κb*
Agah et al. (2019)	*B. bifidum* (Bla/016P/M), *L. acidophilus*	*ifn-γ, il-10*
An et al. (2019)	*L. rhamnosus* KCTC 12202BP	*p53, p21, cyclin b1/cdk1*
An and Ha (2016)	*L. plantarum*	*cd44, 133, 166, aldh1, casp-3*
Baldwin^*^ et al. (2010)	*L. acidophilus* CL1285, *L. casei* LBC80R	*casp-3, p21*
Bassaganya-Riera et al. (2012)	*L. casei, L. plantarum, L. bulgaricus, L. acidophilus, B. longum, B. breve, B. infantis, S. thermophilus*	*tnf-α, angiostatin, ppar c, cox-2, il-17, cd36*
Bertkova et al. (2010)	*L. plantarum*	*tnf-α, il-6*
Borowicki et al. (2011)	*L. rhamnosus* GG (LGG), *B. lactis* Bb12 (Bb12)	*p21, dr5, wnt2b*
Chen et al. (2009)	*S. boulardii*	*erk1/2, mek1/2, her-2, her-3, igf-1r, egfr, erk, mek, camkii* kinases*, pkc, egfr, igf1r, her-2, her-3, akt*
Chen et al. (2015)	*B. subtilis* (ATCC 23857), *C. butyricum* (ATCC 19398)	*tlr4, myd88, p21, il-22, survivin, nf-κb, erk*
Chen et al. (2017)	*L. johnsonii* BCRC17010, *L. delbrueckii* subsp. *bulgarius* BCRC10696, *L. salivarius* BCRC14759, *L. reuteri* BCRC14625, *L. brevis* PM150, *L. plantarum* PM153, *L. brevis* PM177	*bax, bcl-2, ldh, no*
Chondrou et al. (2018)	*L. paracasei* K5, *L. casei* ATCC 393, *L. rhamnosus* GG ATCC 53103	*bcl-2, bcl-xl, bak, bax, bad, b-actin*
Chung et al. (2017)	VSL#3: *L. acidophilus, L. plantarum, L. casei, L. delbrueckii* subsp. *bulgaricus, B. breve, B. longum, B. infantis* and *S. salivarius* subsp. *thermophilus*	*anti-pampk, anti-ampk, anti p-perk1/2, anti-perk1/2, anti-pakt, anti-akt, anti-cyclin d1, anti- bcl-2*
Chung et al. (2019)	*E. faecalis* strain KH2, *Proteus mirabilis* (ATCC 12453)	*il-1β, casp-1*
Cousin et al. (2016)	*Propionibacterium freudenreichii* ITG P9	*casp-8, cyt c, hsc70, p21, parp-1, flip, casp-3, casp-9, bax, bak, bcl-xl, mcl-1, xiap, histone h3*
Del Carmen et al. (2016)	*S. thermophilus* CRL807, *L. delbrueckii* subsp. *bulgaricus* CRL864	*il-10, il-17, mcp-1, tnf-α*
Djaldetti and Bessler (2017)	*S. thermophilus, L. rhamnosus, L. acidophilus, L. casei, B. bifidum, B. longum*	*tnf-α, il-1β, il-6, ifnγ, il-10, il-1ra*
Ewaschuk et al. (2006)	*L. casei, L. plantarum, L. acidophilus, L. delbrueckii* subsp. *bulgaricus, B. infantis, B. breve, B. longum, S. salivarius* subsp. *thermophilus*	*pparg*
Fahmy et al. (2019)	*B. longum* (BL)	*il-1β, il6, mir-21a, mir-155, mir-145, mir-15a, nf-κb*
Foo et al. (2011)	*B. longum* BCRC 910051, *L. gasseri* BCRC 910197	*pcna, p27, cyclin a, cyclin b, cyclin e, cdc-2, cdk-2*
Gamallat et al. (2016)	*L. rhamnosus* GG CGMCC 1.2134 (LGG)	*tnf-α, bcatenin, nfkb, p65, casp-3, cox-2, bax, bcl-2, inos, vegfa, p53, b-actin*
Golkhalkhali et al. (2018)	*L. acidophilus* BCMCR 12130, *L. casei* BCMCR 12313, *L. lactis* BCMCR 12451, *B. bifidum* BCMCR 02290, *B. longum* BCMCR 02120, *B. infantis* BCMCR 02129	*crp, il-6, tnf-α*
Hadad et al. (2019)	*L. rhamnosus*	*tnf-α, il-6*
Heydari et al. (2019)	*B. bifidum* (Bla/016P/M), *L. acidophilus* (LA)	*mir-135b, mir-26b, mir-18a-3p, mir-155-5p, apc, pten, kras, pu.1, b-actin*
Ko et al. (2007)	*L. plantarum* (ATCC 8014)	*tnf-α, il-8, erk*
Konishi et al. (2016)	*L. rhamnosus* GG ATCC53103, *L. casei* ATCC334, *L. coryniformis* ATCC25600, *L. fermentis* ATCC23271	*ddit3, e p44/ 42 mapk (erk), akt, jnk, p38mapk, gsk3b, casp-3, parp*
Kuugbee et al. (2016)	*L. acidophilus, B. bifidum, B. infantum*	*muc2, zo-1, tlr2, tlr4, casp-3, cox-2, b-catenin*
Liu et al. (2013)	*L. plantarum* CGMCC no.1258, *L. acidophilus*-11, *B. longum*-88	*p38 mapk*
Ma et al. (2010)	*B. polyfermenticus*	*erbb2, erbb3, e2f, h2a, il-8*
Mendes et al. (2018)	*L. acidophilus, L. rhamnosus, B. bifidum*	*il-6, il-1β, tnf, il-10, il-4, il-13, tgfβ1*
Mi et al. (2017)	*B. infantis*	*tnf-α, il- 1β, il-6, t-bet, il-2, il-10, tgf-β, il-12, ifn-γ, il-23, il-21, il-17, rorγt, foxp3, gapdh*
Norouzi et al. (2018)	*L. lactis* subsp. *lactis*	*cea, ceam6, mmp2f*
		
Rong et al. (2019)	*L. helveticus* NS8	*tnf-α, il-8, il-10, nf-κb*,
		
Song et al. (2018)	*B. longum, L. acidophilus, E. faecalis*	*cxcl1, cxcl2, cxcl3, cxcl5, tnfa, il1b, il6, ptgs1*
Lee et al. (2020)	*L. fermentum*	*bax, bak, noxa, parp1, bcl-xl, p-iκbα*
Do et al. (2016)	VSL#3: *L. acidophilus, L. plantarum, L. casei, L. delbrueckii* subsp. *bulgaricus, B. breve, B. longum, B. infantis* and *S. salivarius* subsp. *thermophilus*	*tnf-α, il-6, mcp-1, ccl2, il-10, il-11, il-17, il-22, arginase-1*
Yue et al. (2020)	*L. plantarum* YYC-3	*tnf-α, il-6, il-17f, il-22, cyclin-d1, c-myc, icam1, vcam1*
Kim et al. (2020)	*L. johnsonii, L. reuteri, L. rhamnosus* GG	*il-10, ifn γ, il-17, il-12, p40, tbet, gata3, rorc, foxp3*
Dong et al. (2020)	*L. salivarius* (LS) Ren	*akt, jnk, erk, cyclind1, cox2*
Tiptiri-Kourpeti et al. (2016)	*L. casei* ATCC 393	*trail, cyclin d1, birc5a*
Walia et al. (2015)	*L. plantarum* (AdF10), *L. rhamnosus* GG (LGG)	*cox-1, cox-2*
Chen et al. (2020)	*C. butyricum* (ATCC 19398)	*hgpr41, hgpr43, hgpr109a, hp21waf1, hgapdh, mgpr41, mgpr43, mgpr109a, mgapdh, cyclin d1, c-myc*,
Saito et al. (2019)	*L. casei* strain Shirota and *B. breve* strain Yakult	*il-6, stat-3, cox-2, tnf-α, eil-6, stat3, nf-κb, pge-2, cox-2, tnf-α*

### Generation of Candidate Gene-Association Network of Probiotic–Inflammation–CRC Axis

Agilent Literature Search 3.1.1 beta (LitSearch version 2.69 plug-in of Cytoscape 3.7.1) included all the 135 training genes for text-mining–based association network development. The generated association network included the candidate genes from unstructured texts related to inflammation that causes CRC oncogenesis. The primary association network showed 504 nodes and 1,423 edges using training genes as query terms in Cytoscape. Supplementary Figure 2 depicts the association network topology with the training genes (highlighted) related to CRC-promoting gut inflammation. The details of the network parameter statistics are described in the supplementary file (Supplementary Table 1). Such a system biology approach of network analysis using Cytoscape has been conducted to elucidate the pathways of coronary atherosclerosis and in-stent restenosis in humans (Ashley et al., 2006). In addition, the text-mining approach employing Agilent Literature Search with Cytoscape has been adopted by various research groups to show actin dynamics during the post-ejaculatory life of spermatozoa (Bernabo et al., 2016), to understand synergistic mechanisms of therapeutic herbs in the treatment of rheumatic arthritis (Xu et al., 2018), and to investigate the consequences of psychological stress on metabolism and innate immunity in humans (Priyadarshini and Aich, 2012).

### Network Analysis Identified the Promising Gene Clusters Related to Probiotics and CRC Prevention

**Figure 3:**
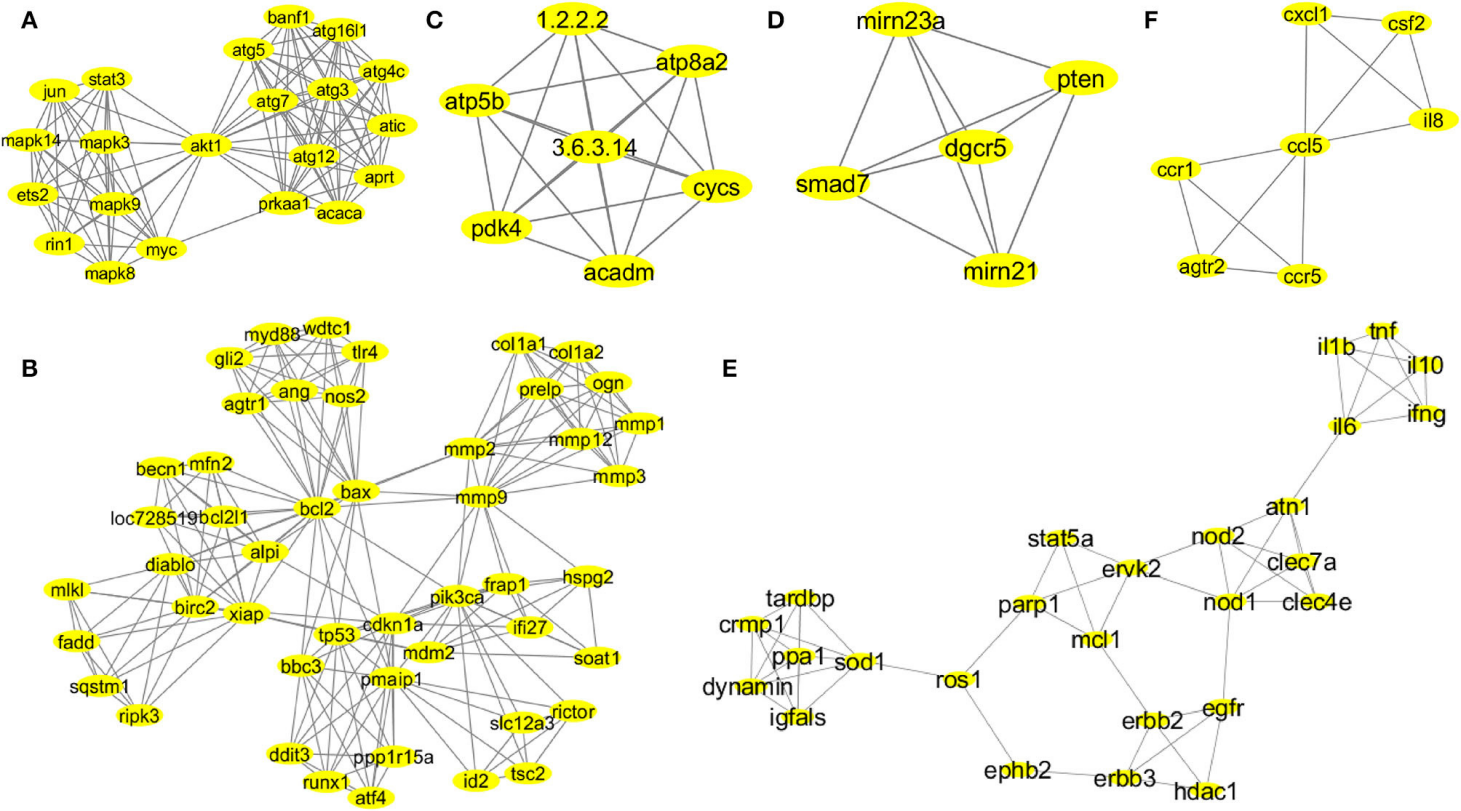
Network topologies of the significant MCODE clusters. MCODE-derived cluster 1: score 11.2, nodes 21 (*mapk14, jun, stat3, mapk8, mapk9, mapk3, myc, ets2, rin1, akt1, atg5, atg7, atg12, atg3, banf1, atg16l1, atg4c, atic, aprt, acaca*, and *prkaa1*), and edges 112 **(A)**. MCODE-derived cluster 2: score 8.894, nodes 48 (*gli2, myd88, wdtc1, tlr4, ang, nos2, agtr1, mlkl, fadd, sqstm1, ripk3, becn1, mfn2, bcl2l1, loc728519, alpi, diablo, birc2, xiap, bcl2, bax, mmp2, mmp9, col1a1, col1a2, ogn, prelp, mmp1, mmp3, mmp12, frap1, pik3ca, ifi27, hspg2, soat1, bbc3, tp53, cdkn1a, mdm2, pmaip1, ddit3, runx1, atf4, pp1r15a, slc12a3, rictor, id2*, and *tsc2*), and edges 209 **(B)**. MCODE-derived cluster 3: score 7, nodes 7 (*atp5b, pdk4, atp8a2, cycs, acadm, 1.2.2.2*, and *3.6.3.14)*, and edges 21 **(C)**. MCODE-derived cluster 4: score 5, nodes 5 (*smad7, pten, dgcr5, mirn21*, and *mirn23a*), and edges 10 **(D)**. MCODE-derived cluster 7: score 4.48, nodes 26 (*il1b, il6, tnf, il10, ifng, atn1, nod2, nod1, clec7a, clec4e, stat5a, parp1, mcl1, ervk2, erbb2, erbb3, egfr, hdac1, ephb2, ros1, sod1, ppa1, dynamin, igfals, tardbp*, and *cmp1*), and edges 56 **(E)**. MCODE-derived cluster 8: score 4, nodes 7 (*ccr1, ccr5, atgr2, il8, csf2, cxcl1*, and *ccl5*), and edges 12 **(F)**.

The MCODE provided functionally significant modules with densely connected nodes in the probiotic–CRC axis. Supplementary Figure 3 shows the topologies of MCODE-derived modules. A total of 43 modules from the first association network were derived by MCODE analysis. The individual cluster scores determined their further selection for functional studies. The MCODE cluster details are depicted in Supplementary Table 2. Among all 43 derived modules, 6 clusters with MCODE score ≥ 4, nodes ≥ 7, edges ≥ 12 and their respective local network topology were chosen for functional annotation using the GeneCards database (version 4.14) and GO enrichment analysis. Module 1 was the highest-scoring module with an MCODE score of 11.2 with 21 nodes and 112 edges, module 2 was the second highest with an MCODE score of 8.894 with 48 nodes and 209 edges, and module 3 was the third highest with a score of 7 with 7 nodes and 21 edges. According to network parameters, the highest-scoring module 1 (MCODE score 11.2) consisted of 21 nodes (*mapk14, jun, stat3, mapk8, mapk9, mapk3, myc, ets2, rin1, akt1, atg5, atg7, atg12, atg3, banf1, atg16l1, atg4c, atic, aprt, acaca*, and *prkaa1*). This was selected as the most significant cluster for CRC-associated inflammation with higher network matrices like closeness centrality, clustering coefficient, and node degree (Figure 3A). Several research groups adopted the MCODE algorithm for module identification by clustering genes of molecular networks (Kim and Kim, 2018; Xu et al., 2018).

### Annotation Profile of the Subclusters Signified a Functional Contribution of Probiotics in the Prevention of Chronic Inflammation Related to CRC

The MCODE analysis identified clusters based on the dense connectivity of nodes among all other closely associated genes in the network (Supplementary Material). The functions of the corresponding nodes or genes of interest and their respective pathways were elucidated by the GeneCards derived functional patterns of the highest-scoring MCODE clusters.

According to MCODE cluster 1 (Figure 3A), the candidate genes were involved in stress-mediated metabolic processes, autophagy, and the induction of apoptosis. Cluster 2 (Figure 3B) candidate genes were involved in response to external stimulus, apoptotic mitochondrial changes, release of cytochrome c, cell proliferation, endoplasmic reticulum stress, regulation of mitochondrial membrane potential and permeability, regulation of *il17* and *il23* production, and NF-KB signaling pathway. Probiotic implementation could play an immense role in regulating apoptosis, stress-induced metabolic pathways, and inflammatory processes. Cluster 3 (Figure 3C) candidate genes were involved in nitrogen biosynthetic, ATP biosynthetic, and metabolic processes. Furthermore, cluster 4 (Figure 3D) candidate genes were involved in the regulation of cell migration and epithelial to mesenchymal transition. Probiotics are reported to modulate host cellular metabolic processes by producing SCFAs and small proteinaceous molecules. Cluster 7 (Figure 3E) candidate genes were associated with cellular response to the bacterium or organic substances, immunomodulatory processes and adaptive immunity, regulation of cytokine and chemokine production, and intracellular protein kinase cascades (JNK, MAPKKK, ERK1, and ERK2). Finally, cluster 8 (Figure 3F) candidate genes were associated with immune response and chemotaxis, inflammatory processes, and cellular metal ion homeostasis.

Probiotics were reported to mediate immunomodulatory activities and were well known for maintaining cellular homeostasis. The functional analysis of the MCODE-derived modules revealed a probable carcinogenic mechanism that activates the inflammatory events leading to CRC development. Thus, the published scientific evidence supported the interconnection of functional profiles of the MCODE clusters in the oncogenic inflammatory processes of CRC, which enlightened us on the unexplored yet promising functional avenues of these genes (see the “Discussion” section).

### GO Enrichment Analysis Assessed the Overrepresentation of GO Categories

In the BiNGO enrichment analysis, the overrepresented or enriched GO terms linked with the candidate genes of MCODE clusters were selected based on the *p*-values (Supplementary Table 3). Our study indicated that cluster 1 (Figure 3A) candidate genes were involved in the pathways related to the cellular response to stress, autophagic vacuole assembly, induction of apoptosis, positive regulation of stress-activated protein kinase cascades (JNK and MAPKKK), and metabolic processes. Therefore, these carcinogenic inflammation-associated physiological events could be targeted by the probiotic implementation for CRC prevention. Cluster 2 (Figure 3B) candidate genes were involved in the cellular response to an external stimulus, apoptotic mitochondrial changes, release of cytochrome c, regulation of cell proliferation, positive regulation of hydrolase and peptidase activity, activation of caspases, response to oxygen levels, endoplasmic reticulum stress, collagen metabolic processes, regulation of *il17* and *il23* production and NF-KB signaling pathway, and positive regulation of phospholipase A2 and phospholipase C activity. Probiotic supplementation could play an immense role in the regulation of TLR4-mediated immune responses, apoptosis, stress-induced metabolic pathways, and inflammatory processes. Cluster 3 (Figure 3C) candidate genes were involved in cellular nitrogen biosynthetic process, ATP biosynthetic and metabolic process, purine ribonucleoside triphosphate biosynthetic process, and fatty acid metabolic processes. Additionally, cluster 4 (Figure 3D) candidate genes were involved in the regulation of cell–cell adhesion, cell–matrix adhesion, epithelial to mesenchymal transition, cell migration, cellular protein localization, and modification. Probiotics are reported to modulate host cellular metabolic processes by producing SCFAs and small proteinaceous molecules. Cluster 7 (Figure 3E) candidate genes were involved in the cell immunomodulatory processes, namely, lymphocyte and leukocyte proliferation, migration and adaptive immunity, regulation of cytokine and chemokine production, cellular response to the bacterium or organic substances, intracellular protein kinase cascades (JNK, MAPKKK, ERK1, and ERK2), regulation of nitric oxide biosynthetic processes, and monooxygenase and oxidoreductase activities. Finally, cluster 8 (Figure 3F) candidate genes were associated with immune response and chemotaxis, cellular metal ion homeostasis, cellular receptor signaling pathways (G-protein jak-stat), and inflammatory processes.

Probiotics and their secretory antimicrobial proteins (AMPs) are well known for their immunomodulatory activities and ability to maintain cellular homeostasis. Thus, the major biological processes that initiate carcinogenic inflammation in gut-epithelium cells were identified by the GO enrichment analysis. These events, therefore, could be the significant cellular domains for the mechanisms involved in the probiotic-mediated CRC prevention.

### Identification of Significantly Enriched Pathways Associated With Probiotics–Inflammation–CRC Axis

Pathway enrichment analyses with 540 candidate genes from the association network were performed using JEPETTO, and 32 significant pathways were obtained (Supplementary Table 4). The XD-score signified a deviation (positive or negative) of all pathways from the average distance. The implication of overlap (Fisher's exact test) between input information and pathways was determined by the *q*-value. The number of overlapping proteins was shown by the overlap/size and compared with the size of the pathway. The threshold value of XD-score was 0.6, and the XD-score and *q*-value indicated the highest XD-score to be 2.33020, as analyzed by the enrichment algorithm. There are 13 cancer pathways (bladder cancer, colorectal cancer, pancreatic cancer, chronic myeloid leukemia, prostate cancer, glioma, endometrial cancer, melanoma, non-small-cell lung cancer, small-cell lung cancer, acute myeloid leukemia, thyroid cancer, and pathways in cancer), 4 infectious disease pathways (leishmaniasis, malaria, Chagas' disease, and shigellosis), 1 neurodegenerative disease [amyotrophic lateral sclerosis (ALS)], 2 developmental pathway (dorso–ventral axis formation and neurotrophin signaling pathway), and 13 signaling pathways including 6 immunomodulatory pathways (NOD-like receptor signaling pathway, toll-like receptor signaling pathway, RIG-I-like receptor signaling pathway, T-cell receptor signaling pathway, Fc epsilon RI signaling pathway, and B-cell receptor signaling pathway), 3 cell growth or death pathways (regulation of autophagy, apoptosis, and p53 signaling pathway), and 3 signal transduction pathways (ErbB signaling pathway, VEGF signaling pathway, and epithelial cell signaling in *Helicobacter pylori* infection) were obtained.

### Molecular Docking Studies Reaffirm the Significant Interactions Between the Key CRC-Causing Inflammatory Proteins With Probiotic-Derived Bacteriocins

Molecular docking is a useful method to simulate intermolecular combinational patterns between the ligands and target proteins to predict possible docking modes and binding affinities. Using *in silico* and *in vitro* binding studies, the interaction of probiotic-derived SCFAs with histone deacetylase (HDAC) has already been established. It is worth mentioning here that the dysregulation of HDAC is associated with several inflammatory conditions (Ho et al., 2017). Such reports encourage us to further study the interaction between bacteriocins and CRC-related proteins.

According to the functional annotation and pathway enrichment analysis of the MCODE clusters, 23 target proteins (candidate genes of cluster 1, cluster 2, cluster 7, and cluster 8) were selected as receptors and 7 bacteriocins derived from different *Lactobacillus* spp. (Holo et al., 1991, 2001; Aymerich et al., 2000; Ferchichi et al., 2001; Yannai, 2012; Yang et al., 2014; Zhao et al., 2016; Anwar et al., 2020) as ligands for docking studies (Supplementary Table 5). All the target proteins were significantly involved in CRC-causing gut inflammation. The lower binding energies of the individual protein–ligand interactions signified a stable binding and greater affinity of the ligand for the respective target proteins (Supplementary Table 6). It was fascinating to observe that, among all, the 12 target proteins (COX-2, PI3K, Caspase 9, IL18R, AKT, TLR4, IKB-A, P38 alpha, mTOR, Caspase 3, Cytochrome C, and ATG4), which were associated with the enriched pathways of the network analysis, showed the most notable binding affinities with studied bacteriocins (Supplementary Figure 4).

**Figure 4:**
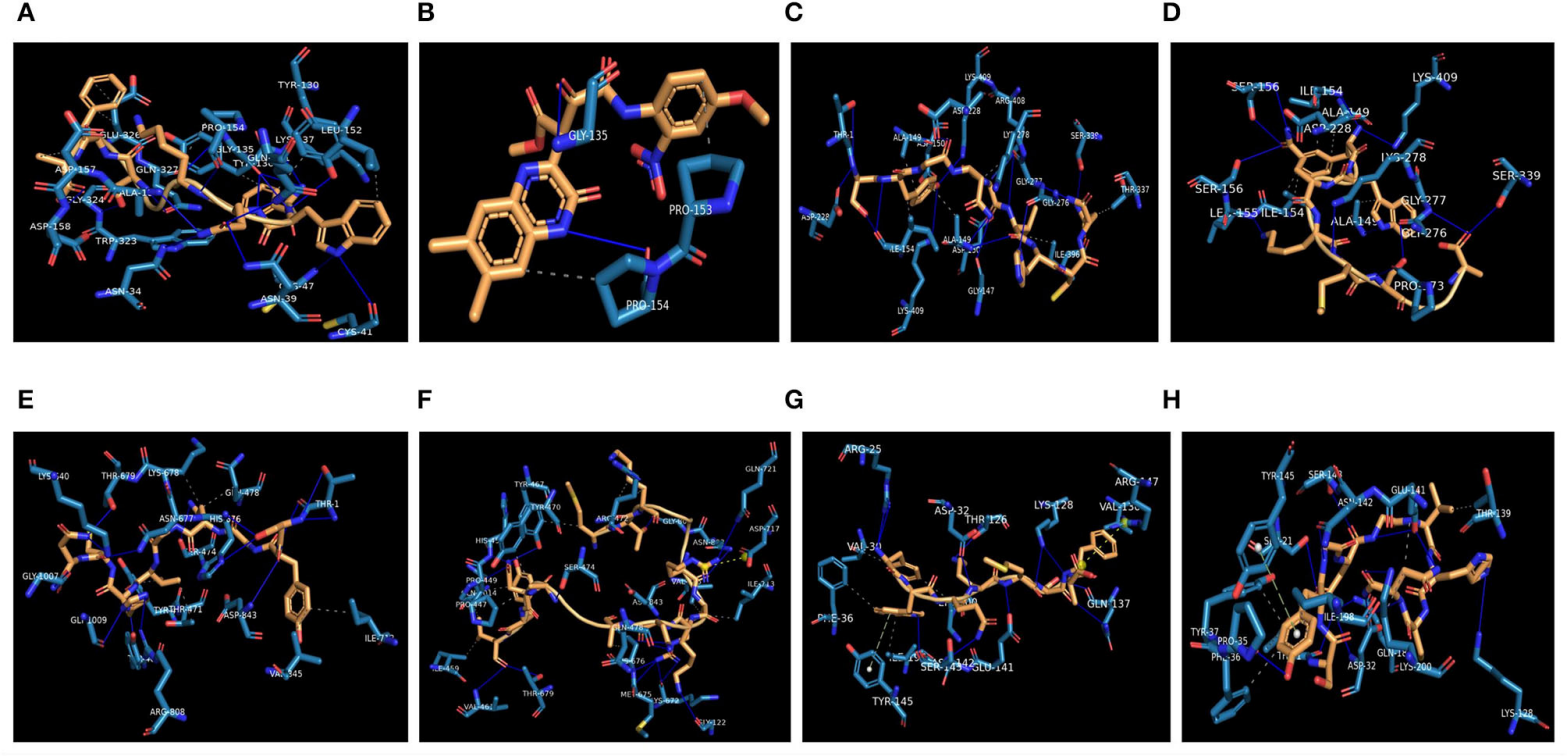
Interaction of CRC-associated proteins and probiotic-derived bacteriocins. COX-2 with Plantaricin JLA-9 **(A)**. COX-2 with lactococcin A **(B)**. CASP9 with Lactococcin mmfii **(C)**. CASP9 with Plantaricin JLA-9 **(D)**. PI3K with Lactococcin mmfii **(E)**. PI3K with Plantaricin W **(F)**. IL18R with Plantaricin JLA-9 **(G)**. IL18R with Lactococcin mmfii **(H)**.

The highest binding affinity (binding energy ≤ −8.3 Kcal/mol) was observed for the interaction between COX-2 and all the 7 bacteriocins (plantaricin JLA-9, plantaricin W, lactococcin A, lactococcin mmfii, bacteriocin 28b, plantaricin D, and plantaricin BN). This result is particularly encouraging since COX-2 is a crucial inflammatory molecule that has been found to be significantly upregulated in CRC (Gupta and Dubois, 2001). According to our docking analysis, plantaricin JLA-9 interacted with the active site of COX-2 (−11 Kcal/mol) with 13 hydrogen-bonds at positions, namely, ASN34, ASN39, CYS41, CYS47, TYR130, GLY135, LYS137, PRO154, ALA156, ASP158, GLY324, GLN327, GLN461, and 7 hydrophobic interactions in the positions, namely, TYR136, LYS137, LEU152, PRO154, ASP157, TRP323, and GLU326 (Figure 4A). COX-2 active site residues GLY135 and PRO154 formed hydrogen bonds and PRO153 and PRO154 interacted with hydrophobic bonds with lactococcin A (−9.6 Kcal/mol) (Figure 4B). Therefore, indeed, probiotic-derived bacteriocins could play a significant role in reducing CRC severity (Table 3).

**Table 3:** Interaction of bacteriocins with COX-2, CASP9, PI3K, and IL18R by molecular docking.

**Ligands**	**Proteins**			
	**Cox2**	**Caspase9**	**PI3K**	**IL18R**
Plantaricin JLA-9	**Hydrogen bond:** ASN34, ASN39, CYS41, CYS47, TYR130, GLY135, LYS137, PRO154, ALA156, ASP158, GLY324, GLN327, GLN461 **Hydrophobic interaction:** TYR136, LYS137, LEU152, PRO154, ASP157, TRP323, GLU326	**Hydrogen bond:** ALA149, ILE154, LEU155, SER156, ASP228, PRO273, GLY276, GLY277, LYS278, SER339, LYS409 **Hydrophobic interaction:** ALA149, ILE154	**Hydrogen bond:** ASN170, SER275, ASN756, ARG818, CYS838, GLU849 **Hydrophobic interaction:** PHE666, ASN822, LEU834, LEU839 **pi-Stacking:** PHE666	**Hydrogen bond:** ARG25, ASP32, THR126, LYS128, GLN137, GLU141, ASN142, SER143 **Hydrophobic interaction:** VAL30, PHE36, VAL130, ILE198, LYS200 **Salt bridge:** ARG147 **pi-stacking:** TYR145
Lactococcin A	**Hydrogen bond:** GLY135, PRO154 **Hydrophobic interaction:** PRO153, PRO154	**Hydrogen bond:** ARG146, LEU155 **Hydrophobic interaction:** ILE154, Lys278 **pi-Cation interaction:** LYS278	**Hydrogen bond:** ASN457, SER474, SER474, GLN475, ASN677 **Hydrophobic interaction:** TRP446, PRO447, LYS678, THR679 **Salt Bridge:** HIS676	**Hydrogen bond:** GLU31, ASP32, GLN124, ASN142 **Hydrophobic interaction:** GLU31, ASP32, THR126, LYS200
Lactococcin mmfii	**Hydrogen bond:** THR1, ASN43, ARG44, TYR122, PHE367, GLN372, LYS468, SER471, LYS473 **Hydrophobic interaction:** PHE64, PHE367, LYS468, PRO542	**Hydrogen bond:** THR1, GLY147, ALA149, ASP150, ILE154, ASP228, GLY276, GLY277, SER339, ARG408, LYS409 **Hydrophobic interaction:** ALA149, ILE154, LYS278, THR337, ILE396	**Hydrogen bond:** THR1, TYR467, SER474, LYS640, HIS676, ASN677, THR679, ARG808, ASP843, GLY1007, GLY1009 **Hydrophobic interaction:** TYR470, THR471, GLN478, LYS678, ILE713, VAL845	**Hydrogen bond:** THR1, GLN18, SER21, ASP32, PRO35, LYS128, GLU141, ASN142, SER143, LYS200 **Hydrophobic interaction:** THR1, PHE36, TYR37, THR139, GLU141, ILE198, LYS200 **pi-Stacking:** TYR145
Plantaricin W	**Hydrogen bond:** ARG44, LYS83, TYR115, ARG120, SER121, TYR122, ILE124, ASP125, SER126, LEU366, GLN370, GLN372, TYR373, LYS532 **Hydrophobic interaction:** ARG44, PHE64, LYS79, LEU80, VAL89, VAL116, TYR122, LEU123, PHE371 **pi-stacking:** TYR115	**Hydrogen bond:** SER144, GLY147, ASN148, LEU155, ALA220, THR270, GLY276, GLY277, LYS278, LEU335, THR337, GLU378, TYR397, PHE412 **Hydrophobic interaction:** ALA141, GLU143, LEU145, LEU151, PRO273, LYS278, TYR397	**Hydrogen bond:** GLY122, HIS450, VAL461, TYR467, ARG472, SER474, GLN478, LYS672, MET675, HIS676, THR679, GLN721, ASN803, GLY804, ASP843, GLN1014 **Hydrophobic interaction:** PRO447, PRO449, ILE459, TYR467, TYR470, ARG472, GLN478, ILE713, VAL845 **Salt bridge:** ASP717	**Hydrogen bond:** ARG39, LYS67, GLU69, ARG104, SER105, ASP110, GLN114, GLY122, LEU144, ASP209, ASN212, VAL214, THR306 **Hydrophobic interaction:** PRO43, GLU69, LYS70, ARG104, PRO107, LEU144, ILE213, VAL214 **Salt bridge:** GLU116, GLU121
Bacteriocin 28b	**Hydrogen bond:** GLY225, VAL228, ASN375, GLY536, ASN537 **Hydrophobic interaction:** LEU145 **pi-stacking:** PHE142	**Hydrogen bond:** ASP228 **Hydrophobic interaction:** ALA149, ILE154	**Hydrogen bond:** SER629, HIS670, ARG818, CYS838 **Hydrophobic interaction:** ARG662 **pi-stacking:** PHE666	**Hydrogen bond:** SER7, SER50, LEU144 **Hydrophobic interaction:** ILE48, ILE71, PRO107
Plantaricin D	**Hydrogen bond:** ASN34, ASN39, CYS47, SER49, VAL132, GLY135, TYR136, GLU322, TRP323, GLN461 **Hydrophobic interaction:** ASN34, LEU152, ALA156, GLU326	**Hydrogen bond:** GLY147, ALA149, ILE154, GLY276, GLY277, LEU335, THR337, SER339, ARG408 **Hydrophobic interaction:** ILE154, LYS278, ILE396	**Hydrogen bond:** ARG4, HIS450, TYR467, SER474, HIS676, ASP843, MET1010 **Hydrophobic interaction:** TRP424, PRO449, GLN478, MET675, HIS676, LYS678, THR679	**Hydrogen bond:** LYS4, LEU5, SER7, HIS109, ASN111, LYS128, ILE129, GLU131 **Hydrophobic interaction:** ILE48, ILE71, VAL130, LYS134, PHE135
Plantaricin BN	**Hydrogen bond:** ASN34, SER49, GLY135, ASP158, TRP323, GLN327 **Hydrophobic interaction:** TYR136, GLU326, GLN327	**Hydrogen bond:** ARG146, GLY147, TYR153, LEU155, Phe412, THR415 **Hydrophobic interaction:** ILE154, LEU155, LYS414 **Salt bridge:** LYS278	**Hydrogen bond:** HIS450, ASN457, ASN677, THR679 **Hydrophobic interaction:** TRP424, TRP446, PRO449 **Salt bridge:** LYS640, ARG80	**Hydrogen bond:** ARG25, ASP32, THR34, TYR37, ASP37, SER143 **Hydrophobic interaction:** THR23, PHE36, TYR37, TYR145, ILE198, LYS200 **Salt bridge:** ARG25, LYS200

Our docking study also indicated that probiotic-derived molecules could have a remarkable strong association with stress-induced kinase PI3K, AKT, P38 alpha, and immunomodulatory IL18R, which contribute to the upregulation of COX-2. Lactococcin mmfii interacted with the initiator Caspase 9 (−9.2 Kcal/mol) with 11 hydrogen bonds and 5 hydrophobic interactions (Figure 4C). Plantaricin JLA-9 also interacted with Caspase 9 (−8.8 Kcal/mol) (Figure 4D; Table 3) with 11 hydrogen bonds and 2 hydrophobic interactions. The docking complex of lactococcin mmfii and PI3K was also found to be substantially strong (−9 Kcal/mol), with 11 amino acids participating in the hydrogen bond formation and 6 residues involved in hydrophobic bond formation (Figure 4E). Plantaricin W interacted with PI3K (−8.5 Kcal/mol) with 16 hydrogen bonds and 9 hydrophobic bonds. The complex was further stabilized by salt bridge interaction with acidic amino acid ASP717 in the active site of PI3K (Figure 4F; Table 3). Furthermore, the immunomodulatory molecule IL18R has been found to interact with plantaricin JLA-9 (−8.4 Kcal/mol) by 8 hydrogen bonds and 5 hydrophobic interactions. The formation of salt bridge interaction with the basic amino acid ARG147 and pi-Stacking interaction with TYR145 increased the stability of the complex (Figure 4G). Lactococcin mmfii also interacted with IL18R (−8.3 Kcal/mol) by forming 10 hydrogen bonds and 7 hydrophobic interactions. The pi-Stacking with TYR145 in the active site of IL18R also escalated the interaction (Figure 4H; Table 3). Therefore, the stable interaction of the bacteriocins with initiator Caspase 9, Caspase 3, Cytochrome C, TLR4, IKB-A, mTOR, and ATG4 could strengthen the role of probiotics in cell survival and antimicrobial pathways (Supplementary Table 7).

## Discussion

The application of probiotics has been recognized as a promising strategy for CRC prevention and treatment. However, decoding the associated molecular mechanisms has been a challenge. To the best of our knowledge, this is the first study that integrates meta-analysis, systematic network, and molecular docking studies to examine the efficacy and mechanisms of probiotic intervention in CRC-associated inflammation in the gut. The meta-analysis rationalizes the use of probiotics in the prevention and treatment of CRC-causing gut inflammation. Network and pathway enrichment analysis, together with molecular docking studies, identifies crucial functional domains of the probiotics–inflammation–CRC axis and provides significant molecular details to unravel the potential mechanisms for probiotic intervention attenuating CRC-related inflammation.

An elevated level of COX-2 is observed at the site of intestinal inflammation, which causes 85% of human colorectal carcinomas (Gupta and Dubois, 2001). Our network analysis with candidate genes of cluster 7 and cluster 8 [Figures 3E,F (*tnf*, *il1b, il6, il10*, etc., *il8, ccr1*), (*ccr5, cxcl, ccl5*, etc.)] indicates inflammatory pathways as the potential domain where the application of probiotics could be effective. Our molecular docking analysis directly shows that probiotic-derived bacteriocins have the highest binding affinity for COX-2. Previously, probiotic and synbiotic formulations have been shown to mitigate the elevated expression of inflammatory COX-2 in *in vitro* and *in vivo* models (Bassaganya-Riera et al., 2012; Greenhalgh et al., 2019). Recently, probiotic-fermented whole grains like germinated brown rice (FGBR) have been shown to reduce increased levels of pro-inflammatory TNFα, IL6, and IL1β in chemically induced CRC rat models (Li et al., 2019b). Therefore, our study beautifully complements the previous study and indicates that probiotics could downregulate the cyclooxygenase pathway by inhibiting COX-2 and reduce the oncogenic inflammation in the epithelium lining.

Induced COX-2 expression is at the nexus of various signaling cascades that are aberrantly regulated in cancer. One of them is cellular oxidative stress (ROS generation) and stress-induced metabolic processes (PI3K/AKT) (Koundouros and Poulogiannis, 2018). The cellular oxidative balance is disturbed by the toxic extracellular stimulus that turns on stress-induced metabolic processes and the host adaptive immune system in the gut epithelial cells. The ionic dysregulation can further induce oxidative nitric oxide synthesis that promotes activation of the NF-κB pathway, MMPs, and COX-2, which are found at an elevated level in the carcinogenic gut inflammation (Koundouros and Poulogiannis, 2018). Such activated inflammatory pathways stimulate the secretion of pro-inflammatory cytokines and chemokines, leading to a chronic inflammatory environment in the colonic epithelium, a hallmark of CRC. Our network analysis with candidate genes of cluster 1, cluster 2, cluster 3, cluster 4, and cluster 7 [Figures 3A–E (*akt, mapk, jun, stat3, myc*, etc.), (*mmps*, etc.), (*pdk4*, etc.), (*smad, pten*, etc.), (*sod, ros, parp, erbb, nod, hdac*)] indicates that probiotics could target these malfunctioned cellular processes to offer a beneficial impact. Indeed probiotics are found to reduce cellular oxidative stress by decreasing nitric oxide (NO) production (Kang et al., 2019). Butyrate, a probiotic-derived metabolite, has been shown to decrease inflammation in colon cancer cells. Probiotics or synbiotics are also reported to reduce pro-inflammatory IL8 secretion and elevate levels of p65-NFκB, p38 MAPK, and COX2 in inflammatory intestinal epithelial cells (IECs). Thus, probiotics are shown to exhibit a promising anti-inflammatory activity through regulating TLR2-mediated NFκB and MAPK signaling pathways in chronic gut inflammation (Li et al., 2019a). Although a recent study showed the antitumor effect of IL18 in the presence of natural killer (NK) cells by the CRC model, direct interaction of IL18 with the probiotic-derived molecules has not been elucidated (Li et al., 2021). Our docking study directly shows the robust interactions of PI3K with lactococcin mmfii (−9 Kcal/mol) and plantaricin W (−8.5 Kcal/mol), AKT with plantaricin JLA-9 (−8.7 Kcal/mol), and bacteriocin 28b (−8.3 Kcal/mol), p38a with lactococcin A (−8.1 Kcal/mol). Thus, probiotic-derived metabolites and bacteriocins could modulate stress-induced metabolic processes and reduce gut inflammation. Molecular docking study also depicts the direct interaction of TLR4 with lactococcin mmfii, lactococcin A, plantaricin JLA-9, and plantaricin W with binding energy ≥-7.8 Kcal/mol and thus strengthens the role of probiotics in modulating the dysregulated immunoactivities (Supplementary Table 6). The binding affinity of the pro-inflammatory cytokine receptor IL18R with plantaricin JLA-9 (−8.4 Kcal/mol) and lactococcin mmfii (−8.3 Kcal/mol), as well as the interaction of TNFR1 with lactococcin A (−8.3 Kcal/mol), strongly indicates the anti-inflammatory role of probiotics. In addition, the strong association of IκBA with lactococcin A and plantaricin JLA-9 with binding energies of −8.4 Kcal/mol and −8.1 Kcal/mol, respectively, also supports the anti-inflammatory effect of probiotics. Therefore, the application of probiotics could lower chronic inflammation in the colonic environment by regulating pathways related to metabolism and immunity.

Our GO and pathway enrichment analysis of cluster 1 (*atg5, atg7, atg3, atg12, atg4c, atg16l1*, etc.) indicates that probiotics could have significant effects on autophagy and apoptosis in CRC. The binding energy of initiator Caspase 9 with lactococcin mmfii (−9.2 Kcal/mol) and plantaricin JLA-9 (−8.8 Kcal/mol) along with executioner Caspase 3 with lactococcin A (−8.3 Kcal/mol) and bacteriocin 28b (−8.1 Kcal/mol) further justifies the potential role of probiotics in inducing apoptosis in CRC cells. Furthermore, it has been shown previously that probiotic-derived secondary metabolites, polyamines, and ferrichrome could prevent CRC by inducing ER-stress mediated and intrinsic apoptotic pathways (Konishi et al., 2016). The recent study on *L. acidophilus*-fermented brown rice (FGBR) also proved to upregulate pro-apoptotic Caspase 3, Bax expression and downregulate anti-apoptotic Bcl2 levels in a chemically induced CRC rat model (Li et al., 2019b). Therefore, our *in silico* findings are aptly matched with the previous experimental studies. Although the function of autophagy in colorectal carcinogenesis is controversial, probiotics are reported to enhance the autophagic vacuole assembly, which could improve the inflammatory predisposition of CRC (Figure 3A) (Lai and Huang, 2019; Zaylaa et al., 2019). The binding interaction of autophagy-related cysteine peptidase ATG4 with bacteriocins supports their interactions. Recall, our molecular docking studies also show strong interactions of PI3K with lactococcin mmfii (−9 Kcal/mol) and plantaricin W (−8.5 Kcal/mol) and AKT with plantaricin JLA-9 (−8.7 Kcal/mol). Therefore, probiotics could regulate PI3K/AKT and caspase pathways for the activation of autophagy and apoptosis in CRC (Fan et al., 2016).

**Figure 5:**
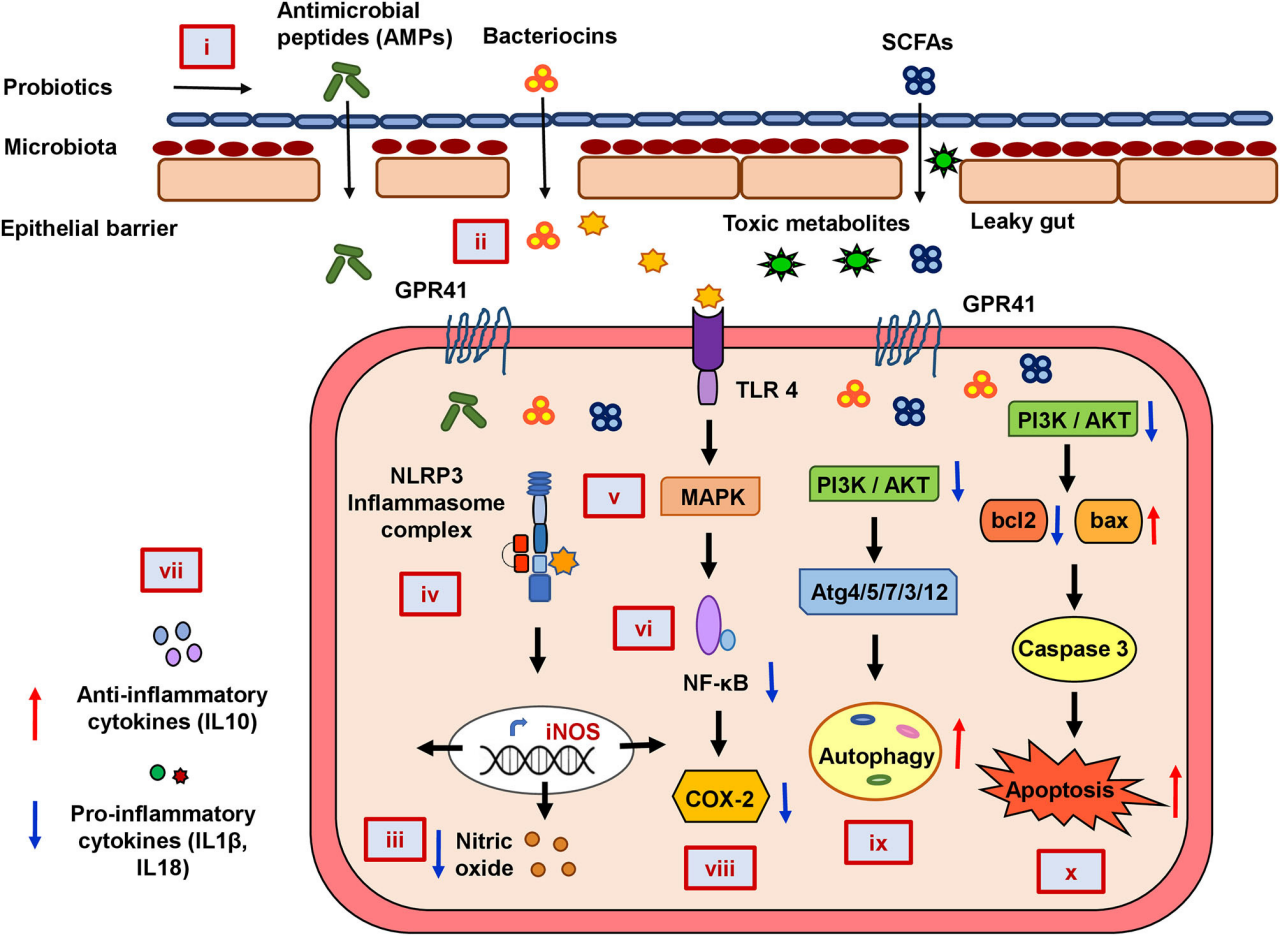
The mechanistic model of the consequences of probiotic application to prevent colorectal cancer promoting gut inflammation. Probiotics strengthen the protective epithelial barrier that prevents the entry of toxic metabolites due to leaky gut (i). Probiotics colonize the epithelial barrier and re-establish the gut equilibrium by producing antimicrobial peptides (AMPs), bacteriocins, and SCFAs that regulate metabolic processes (ii). Probiotics decrease nitric oxide (NO) production and maintain cellular oxidative balance (iii). Probiotics downregulate NLRP3-mediated inflammatory processes (iv), stress-induced MAPK (v), and NF-κB pathways (vi). Probiotics balance host immunity by stimulating anti-inflammatory cytokines and suppressing pro-inflammatory cytokines (vii) and inhibiting COX-2 production (viii). Probiotics downregulate PI3K/AKT and induce autophagy (ix) and apoptotic pathways (x) in CRC.

In summary, our functional annotation of the probiotics–inflammation–CRC gene network and pathway enrichment analysis beautifully corroborate with each other, and our molecular docking analyses further strengthen them by showing the direct interaction between the key targets of the cancer-triggering pathways and the probiotic-derived bacteriocins. Based on our analysis, we propose a rational model of potential mechanisms of probiotic intervention to reduce the severity of chronic inflammation in CRC-pathogenesis (Figure 5). Probiotics and probiotic-derived bacteriocins are known to improve microbial colonization in the GI tract, eliminating pathogens and healing the damaged epithelial barrier. Consumption of probiotics could therefore decrease the entry of toxic metabolites by strengthening the protective epithelial barrier. Probiotic-derived bacteriocins, antimicrobial peptides, and SCFAs could reduce cellular stress by regulating metabolic processes. Probiotics could decrease nitric oxide production and reduce the oxidative stress of the cell. This can downregulate inflammatory (NF-κB and NLRP3) pathways and reduce the secretion of pro-inflammatory chemokines or cytokines. Probiotics and/or bacteriocins may balance anti- and pro-inflammatory cytokines and modulate the expression of COX-2. Finally, probiotic-derived metabolites and proteins could regulate PI3K/AKT and caspase pathways to promote autophagy and apoptosis in the CRC.

## Conclusion

Our systematic network-meta-analysis and *in silico* molecular docking studies provide the key functional domains with their targets and a potential mechanism for probiotic intervention in gut inflammation and CRC. Probiotic-derived metabolites could regulate metabolic processes and reduce cellular oxidative stress. The resulting downregulation of inflammatory pathways could reduce the secretion of pro-inflammatory molecules. Probiotics/probiotic-derived bacteriocins could control pro- and anti-inflammatory cytokine balance and inhibit the expression of COX-2. Finally, bacteriocins and metabolites could also induce autophagy and apoptosis in CRC by regulating PI3K/Akt and caspase pathways. Thus, the probiotic intervention involves multiple biological processes and signaling cascades, yet metabolism and immune pathways could be the dominant targets for probiotic-mediated CRC prevention and treatment. However, detailed experimental studies on these proposed mechanisms would confirm them further. Unraveling the mechanism thus could benefit in developing strategies to tackle the disease efficiently.

## Data Availability Statement

The original contributions presented in the study are included in the article/Supplementary Material, further inquiries can be directed to the corresponding author.

## Author Contributions

SP performed all analyses, including network and meta-analysis, and drafted the manuscript. SS made the tables and helped in drafting the manuscript. NS did the molecular docking studies with BP and SN. AR conceived the study, designed the approach, evaluated the data, and involved in manuscript preparation. All authors reviewed and edited the manuscript.

## Funding

This study was partially supported by the intramural funding provided by IIT Bhubaneswar. SP and SS received institutional fellowships from IIT Bhubaneswar. BP was supported by an endowment grant to IIT Bhubaneswar from the Dr. Dash Foundation, USA.

## Conflict of Interest

The authors declare that the research was conducted in the absence of any commercial or financial relationships that could be construed as a potential conflict of interest.

## Publisher's Note

All claims expressed in this article are solely those of the authors and do not necessarily represent those of their affiliated organizations, or those of the publisher, the editors and the reviewers. Any product that may be evaluated in this article, or claim that may be made by its manufacturer, is not guaranteed or endorsed by the publisher.

## Abbreviations

CRC: colorectal cancer
MCODE: molecular complex detection
ER: endoplasmic reticulum
GI: gastrointestinal
FAO: Food and Agriculture Organization
WHO: World Health Organization
TLR, toll-like receptor, ROS: reactive oxygen species
IL: interleukin.

## References

[B1] Agah S. Alizadeh A. M. Mosavi M. Ranji P. Khavari-Daneshvar H. Ghasemian F.. (2019). More protection of *Lactobacillus Acidophilus* than *bifidobacterium bifidum* probiotics on azoxymethane-induced mouse colon cancer. Probiotics Antimicrob Prot. 11, 857–864. 10.1007/S12602-018-9425-8 29682678

[B2] An B. C. Hong S. Park H. J. Kim B.-K. Ahn J. Y. Ryu Y.. (2019). Anti-colorectal cancer effects of probiotic-derived P8 protein. Genes 10, 624–636. 10.3390/Genes10080624 31430963 PMC6723380

[B3] An J. Ha E.-M. (2016). Combination therapy of *Lactobacillus Plantarum* supernatant and 5-fluouracil increases chemosensitivity in colorectal cancer cells. J. Microbiol. Biotechnol. 26, 1490–1503. 10.4014/Jmb.1605.05024 27221111

[B4] Anwar F. Altayb H. N. Al-Abbasi F. A. Al-Malki A. L. Kamal M. A. Kumar V. (2020). Antiviral effects of probiotic metabolites on Covid-19. J. Biomol. Struct. Dyn. 39, 4175–4184. 10.1080/07391102.2020.1775123 32475223 PMC7298884

[B5] Ashley E. A. Ferrara R. King J. Y. Vailaya A. Kuchinsky A. He X.. (2006). Network analysis of human in-stent restenosis. Circulation 114, 2644–2654. 10.1161/Circulationaha.106.637025 17145989

[B6] Aymerich M. Garriga M. Monfort J. Nes I. Hugas M. (2000). Bacteriocin-producing *lactobacilli* in spanish-style fermented sausages: characterization of bacteriocins. Food Microbiol. 17, 33–45. 10.1006/Fmic.1999.0275

[B7] Bader G. D. Hogue C. W. (2003). An automated method for finding molecular complexes in large protein interaction networks. BMC Bioinform. 4, 1–27. 10.1186/1471-2105-4-2 12525261 PMC149346

[B8] Bajramagic S. Hodzic E. Mulabdic A. Holjan S. Smajlovic S. V. Rovcanin A. (2019). Usage of probiotics and its clinical significance at surgically treated patients sufferig from colorectal carcinoma. Med. Archiv. 73, 316–320. 31819304 10.5455/medarh.2019.73.316-320 PMC6885229

[B9] Baldwin* C. Millette,* M. Oth D. Ruiz M. T. Luquet F.-M. Lacroix M. (2010). Probiotic *Lactobacillus Acidophilus* and *L. Casei* mix sensitize colorectal tumoral cells to 5-fluorouracil-induced apoptosis. Nutr. Cancer 62, 371–378. 10.1080/01635580903407197 20358475

[B10] Bassaganya-Riera J. Viladomiu M. Pedragosa M. De Simone C. Hontecillas R. (2012). Immunoregulatory mechanisms underlying prevention of colitis-associated colorectal cancer by probiotic bacteria. Plos ONE 7, 1–8. 10.1371/Journal.Pone.0034676 22511958 PMC3325233

[B11] Bernabo N. Ordinelli A. Ramal Sanchez M. Mattioli M. Barboni B. (2016). Networks models of actin dynamics during spermatozoa postejaculatory life: a comparison among human-made and text mining-based models. Biomed. Res. Int. 2016, 1–8. 10.1155/2016/9795409 27642606 PMC5013236

[B12] Bertkova I. Hijova E. Chmelarova A. Mojzisova G. Petrasova D. Strojny L.. (2010). The effect of probiotic microorganisms and bioactive compounds on chemically induced carcinogenesis in rats. Neoplasma 57, 422–428. 10.4149/Neo_2010_05_422 20568896

[B13] Borowicki A. Michelmann A. Stein K. Scharlau D. Scheu K. Obst U.. (2011). Fermented wheat aleurone enriched with probiotic strains lgg and bb12 modulates markers of tumor progression in human colon cells. Nutr. Cancer 63, 151–160. 10.1080/01635581.2010.516874 21161821

[B14] Chen C.-C. Lin W.-C. Kong M.-S. Shi H. N. Walker W. A. Lin C.-Y.. (2012). Oral Inoculation of probiotics *Lactobacillus Acidophilus* ncfm suppresses tumour growth both in segmental orthotopic colon cancer and extra-intestinal tissue. Br. J. Nutr. 107, 1623–1634. 10.1017/S0007114511004934 21992995

[B15] Chen D. Jin D. Huang S. Wu J. Xu M. Liu T.. (2020). *Clostridium Butyricum*, a butyrate-producing probiotic, inhibits intestinal tumor development through modulating wnt signaling and gut microbiota. Cancer Lett. 469, 456–467. 10.1016/J.Canlet.2019.11.019 31734354

[B16] Chen E. Xu X. Liu T. (2018). Hereditary nonpolyposis colorectal cancer and cancer syndromes: recent basic and clinical discoveries. J. Oncol. 2018, 1–11. 10.1155/2018/3979135 29849630 PMC5937448

[B17] Chen X. Fruehauf J. Goldsmith J. D. Xu H. Katchar K. K. Koon H. W.. (2009). *Saccharomyces Boulardii* inhibits egf receptor signaling and intestinal tumor growth in apcmin mice. Gastroenterology 137, 914–923. 10.1053/J.Gastro.2009.05.050 19482027 PMC2777664

[B18] Chen Z.-F. Ai L.-Y. Wang J.-L. Ren L.-L. Yu Y.-N. Xu J.. (2015). Probiotics *Clostridium Butyricum* and *Bacillus Subtilis* ameliorate intestinal tumorigenesis. Fut. Microbiol. 10, 1433–1445. 10.2217/Fmb.15.66 26346930

[B19] Chen Z.-Y. Hsieh Y.-M. Huang C.-C. Tsai C.-C. (2017). Inhibitory effects of probiotic *lactobacillus* on the growth of human colonic carcinoma cell line Ht-29. Molecules 22, 1–12. 10.3390/Molecules22010107 28075415 PMC6155858

[B20] Chondrou P. Karapetsas A. Kiousi D. Tsela D. Tiptiri-Kourpeti A. Anestopoulos I.. (2018). *Lactobacillus Paracasei* K5 displays adhesion, anti-proliferative activity and apoptotic effects in human colon cancer cells. Benef. Microbes 9, 975–983. 10.3920/Bm2017.0183 30353740

[B21] Chung E.-J. Do E.-J. Kim S.-Y. Cho E. A. Kim D.-H. Pak S.. (2017). Combination of metformin and Vsl# 3 additively suppresses western-style diet induced colon cancer in mice. Eur. J. Pharmacol. 794, 1–7. 10.1016/J.Ejphar.2016.11.012 27845068

[B22] Chung I.-C. Ouyang C.-N. Yuan S.-N. Lin H.-C. Huang K.-Y. Wu P.-S.. (2019). Pretreatment with a heat-killed probiotic modulates the nlrp3 inflammasome and attenuates colitis-associated colorectal cancer in mice. Nutrients 11, 516–532. 10.3390/Nu11030516 30823406 PMC6471765

[B23] Conlon M. A. Bird A. R. (2014). The impact of diet and lifestyle on gut microbiota and human health. Nutrients 7, 17–44. 10.3390/Nu7010017 25545101 PMC4303825

[B24] Cousin F. J. Jouan-Lanhouet S. Théret N. Brenner C. Jouan E. Le Moigne-Muller G.. (2016). The probiotic *Propionibacterium freudenreichii* as a new adjuvant for trail-based therapy in colorectal cancer. Oncotarget 7, 7161–7178. 10.18632/Oncotarget.6881 26771233 PMC4872776

[B25] Cruz B. Duarte V. Giacomini A. Corich V. De Paula S. O. Fialho L.. (2020). Synbiotic Vsl-3 and yacon-based product modulate the intestinal microbiota and prevent the development of pre-neoplastic lesions in a colorectal carcinogenesis model. Appl. Microbiol. Biotechnol. 104, 8837–8857. 10.1007/S00253-020-10863-X 32902682

[B26] Daniluk U. Alifier M. Kaczmarski M. (2012). Probiotic-induced apoptosis and its potential relevance to mucosal inflammation of gastrointestinal tract. Adv. Med. Sci. 57, 175–182. 10.2478/V10039-012-0025-7 22968339

[B27] Dekker E. Tanis P. J. Vleugels J. L. A. Kasi P. M. Wallace M. B. (2019). Colorectal cancer. Lancet 394, 1467–1480. 10.1016/S0140-6736(19)32319-0 31631858

[B28] Del Carmen S. De Moreno De Leblanc A. Leblanc J. (2016). Development of a potential probiotic yoghurt using selected anti-inflammatory lactic acid bacteria for prevention of colitis and carcinogenesis in mice. J. Appl. Microbiol. 121, 821–830. 10.1111/Jam.13213 27341191

[B29] Delia P. Sansotta G. Donato V. Frosina P. Messina G. De Renzis C.. (2007). Use of probiotics for prevention of radiation-induced diarrhea. World J. Gastroenterol. 13, 912–915. 10.3748/Wjg.V13.I6.912 17352022 PMC4065928

[B30] Deol P. K. Khare P. Bishnoi M. Kondepudi K. K. Kaur I. P. (2018). Coadministration of ginger extract-*Lactobacillus Acidophilus* (Cobiotic) reduces gut inflammation and oxidative stress via downregulation of cox-2, I-Nos, And C-Myc. Phytother. Res. 32, 1950–1956. 10.1002/Ptr.6121 29876980

[B31] Djaldetti M. Bessler H. (2017). Probiotic Strains modulate cytokine production and the immune interplay between human peripheral blood mononucear cells and colon cancer cells. Fems Microbiol. Lett. 364, 1–5. 10.1093/Femsle/Fnx014 28104778

[B32] Do E. J. Hwang S. W. Kim S. Y. Ryu Y. M. Cho E. A. Chung E. J.. (2016). Suppression of colitis-associated carcinogenesis through modulation of il-6/stat3 pathway by balsalazide and Vsl# 3. J. Gastroenterol. Hepatol. 31, 1453–1461. 10.1111/Jgh.13280 26711554

[B33] Dong H. Rowland I. Yaqoob P. (2012). Comparative effects of six probiotic strains on immune function in vitro. Br. J. Nutr. 108, 459–470. 10.1017/S0007114511005824 22054064

[B34] Dong Y. Zhu J. Zhang M. Ge S. Zhao L. (2020). Probiotic *Lactobacillus Salivarius* ren prevent dimethylhydrazine-induced colorectal cancer through protein kinase b inhibition. Appl. Microbiol. Biotechnol. 104, 7377–7389. 10.1007/S00253-020-10775-W 32666185

[B35] Ewaschuk J. B. Walker J. W. Diaz H. Madsen K. L. (2006). Bioproduction of conjugated linoleic acid by probiotic bacteria occurs in vitro and in vivo in mice. J. Nutr. 136, 1483–1487. 10.1093/Jn/136.6.1483 16702308

[B36] Fahmy C. A. Gamal-Eldeen A. M. El-Hussieny E. A. Raafat B. M. Mehanna N. S. Talaat R. M.. (2019). *Bifidobacterium Longum* suppresses murine colorectal cancer through the modulation of oncomirs and tumor suppressor mirnas. Nutr. Cancer. 71, 688–700. 10.1080/01635581.2019.1577984 30862187

[B37] Fan X.-J. Wang Y. Wang L. Zhu M. (2016). Salidroside induces apoptosis and autophagy in human colorectal cancer cells through inhibition of Pi3k/Akt/Mtor pathway. Oncology Reports 36, 3559–3567. 10.3892/Or.2016.5138 27748934

[B38] Femia A. P. Luceri C. Dolara P. Giannini A. Biggeri A. Salvadori M.. (2002). Antitumorigenic activity of the prebiotic inulin enriched with oligofructose in combination with the probiotics *Lactobacillus Rhamnosus* and *Bifidobacterium Lactis* on azoxymethane-induced colon carcinogenesis in rats. Carcinogenesis 23, 1953–1960. 10.1093/Carcin/23.11.1953 12419846

[B39] Ferchichi M. Frère J. Mabrouk K. Manai M. (2001). Lactococcin mmfii, a novel class iia bacteriocin produced by *Lactococcus lactis* mmfii, isolated from a tunisian dairy product. Fems Microbiol. Lett. 205, 49–55. 10.1111/J.1574-6968.2001.Tb10924.X 11728715

[B40] Flesch A. T. Tonial S. T. Contu P. D. C. Damin D. C. (2017). A administração perioperatória de simbióticos em pacientes com câncer colorretal reduz a incidência de infecções pós-operatórias: ensaio clínico randomizado duplo-cego. Rev. Col. Bras. Cir. 44, 567–573. 10.1590/0100-69912017006004 29267553

[B41] Foo N.-P. Ou Yang H. Chiu H.-H. Chan H.-Y. Liao C.-C. Yu C.-K.. (2011). Probiotics prevent the development of 1, 2-dimethylhydrazine (dmh)-induced colonic tumorigenesis through suppressed colonic mucosa cellular proliferation and increased stimulation of macrophages. J. Agric. Food Chem. 59, 13337–13345. 10.1021/Jf203444d 22049926

[B42] Gamallat Y. Meyiah A. Kuugbee E. D. Hago A. M. Chiwala G. Awadasseid A.. (2016). *Lactobacillus Rhamnosus* induced epithelial cell apoptosis, ameliorates inflammation and prevents colon cancer development in an animal model. Biomed Pharmacother. 83, 536–541. 10.1016/J.Biopha.2016.07.001 27447122

[B43] George Kerry R. Patra J. K. Gouda S. Park Y. Shin H. S. Das G. (2018). Benefaction of probiotics for human health: a review. J. Food Drug. Anal. 26, 927–939. 10.1016/J.Jfda.2018.01.002 29976412 PMC9303019

[B44] Golkhalkhali B. Rajandram R. Paliany A. S. Ho G. F. Wan Ishak W. Z. Johari C. S.. (2018). Strain-specific probiotic (microbial cell preparation) and omega-3 fatty acid in modulating quality of life and inflammatory markers in colorectal cancer patients: a randomized controlled trial. Asia Pac. J. Clin. Oncol. 14, 179–191. 10.1111/Ajco.12758 28857425

[B45] Greenhalgh K. Ramiro-Garcia J. Heinken A. (2019). Integrated in vitro and in silico modeling delineates the molecular effects of a synbiotic regimen on colorectal-cancer-derived cells. Cell Rep. 27, 1621–1632. 10.1016/J.Celrep.2019.04.001 31042485

[B46] Gupta R. A. Dubois R. N. (2001). Colorectal cancer prevention and treatment by inhibition of cyclooxygenase-2. Nat. Rev. Cancer 1, 11–21. 10.1038/35094017 11900248

[B47] Hadad S. Hazmi B. Alhebshi A. Aldahlawi A. M. Bassam R. (2019). *Lactobacillus Rhamnosus* enhances the immunological antitumor effect of 5-fluorouracil against colon cancer. Pak J. Biol. Sci. 22, 597–606. 10.3923/Pjbs.2019.597.606 31930859

[B48] Hatakka K. Holma R. El-Nezami H. Suomalainen T. Kuisma M. Saxelin M.. (2008). The Influence of *Lactobacillus rhamnosus* lc705 together with *Propionibacterium Freudenreichii* Ssp. *S*hermanii Js on potentially carcinogenic bacterial activity in human colon. Int. J. Food Microbiol. 128, 406–410. 10.1016/J.Ijfoodmicro.2008.09.010 18945506

[B49] Heydari Z. Rahaie M. Alizadeh A. M. Agah S. Khalighfard S. Bahmani S. (2019). Effects of *Lactobacillus Acidophilus* And *Bifidobacterium Bifidum* probiotics on the expression of micrornas 135b, 26b, 18a and 155, and their involving genes in mice colon cancer. Probiotics Antimicrob. Proteins 11, 1155–1162. 10.1007/S12602-018-9478-8 30311185

[B50] Ho R. H. Chan J. C. Y. Fan H. Kioh D. Y. Q. Lee B. W. Chan E. C. Y. (2017). In silico and in vitro interactions between short chain fatty acids and human histone deacetylases. Biochemistry 56, 4871–4878. 10.1021/Acs.Biochem.7b00508 28809557

[B51] Holo H. Jeknic Z. Daeschel M. Stevanovic S. Nes I. F. (2001). Plantaricin W from *Lactobacillus Plantarum* belongs to a new family of two-peptide lantibiotics. Microbiology 147, 643–651. 10.1099/00221287-147-3-643 11238971

[B52] Holo H. Nilssen Ø. Nes I. (1991). Lactococcin A, A New Bacteriocin From *Lactococcus Lactis* Subsp. cremoris: isolation and characterization of the protein and its gene. J. Bacteriol. 173, 3879–3887. 10.1128/Jb.173.12.3879-3887.1991 1904860 PMC208020

[B53] Huang L. Chiau J.-S. C. Cheng M.-L. Chan W.-T. Jiang C.-B. Chang S.-W.. (2019). Scid/nod mice model for 5-fu induced intestinal mucositis: safety and effects of probiotics as therapy. Pediatr. Neonatol. 60, 252–260. 10.1016/J.Pedneo.2018.07.007 30150027

[B54] Irecta-Nájera C. A. Del Rosario Huizar-López M. Casas-Solís J. Castro-Félix P. Santerre A. (2017). Protective effect of *Lactobacillus Casei* on dmh-induced colon carcinogenesis in mice. Probiotics Antimicrob. Proteins 9, 163–171. 10.1007/S12602-017-9253-2 28316010

[B55] Ishikawa H. Akedo I. Otani T. Suzuki T. Nakamura T. Takeyama I.. (2005). Randomized trial of dietary fiber and *lactobacillus casei* administration for prevention of colorectal tumors. Int. J. Cancer 116, 762–767. 10.1002/Ijc.21115 15828052

[B56] Jandhyala S. M. Talukdar R. Subramanyam C. Vuyyuru H. Sasikala M. Nageshwar Reddy D. (2015). Role of the normal gut microbiota. World J. Gastroenterol. 21, 8787–8803. 10.3748/Wjg.V21.I29.8787 26269668 PMC4528021

[B57] Kaeid Sharaf L. Shukla G. (2018). Probiotics (*Lactobacillus Acidophilus* And *Lactobacillus Rhamnosus* Gg) in conjunction with celecoxib (selective cox-2 inhibitor) modulated dmh-induced early experimental colon carcinogenesis. Nutr. Cancer 70, 946–955. 10.1080/01635581.2018.1490783 30183370

[B58] Kahouli I. Tomaro-Duchesneau C. Prakash S. (2013). Probiotics in colorectal cancer (crc) with emphasis on mechanisms of action and current perspectives. J. Med. Microbiol. 62, 1107–1123. 10.1099/Jmm.0.048975-0 23558140

[B59] Kang C. H. Han S. H. Kim J. S. Kim Y. Jeong Y. Park H. M.. (2019). Inhibition of nitric oxide production, oxidative stress prevention, and probiotic activity of lactic acid bacteria isolated from the human vagina and fermented food. Microorganisms 7, 109–119. 10.3390/Microorganisms7040109 31018570 PMC6518130

[B60] Karimi Ardestani S. Tafvizi F. Tajabadi Ebrahimi M. (2019). Heat-killed probiotic bacteria induce apoptosis of ht-29 human colon adenocarcinoma cell line via the regulation of bax/bcl2 and caspases pathway. Hum. Exp. Toxicol. 38, 1069–1081. 10.1177/0960327119851255 31117840

[B61] Khan S. Moore R. J. Stanley D. Chousalkar K. K. (2020). The gut microbiota of laying hens and its manipulation with prebiotics and probiotics to enhance gut health and food safety. Appl. Environ. Microbiol. 86, E00600–00620. 10.1128/Aem.00600-20 32332137 PMC7301851

[B62] Kim H. Kim Y. M. (2018). Pan-Cancer analysis of somatic mutations and transcriptomes reveals common functional gene clusters shared by multiple cancer types. Sci. Rep. 8, 6041–6055. 10.1038/S41598-018-24379-Y 29662161 PMC5902616

[B63] Kim J.-H. Kordahi M. C. Chac D. Depaolo R. W. (2020). Toll-like receptor-6 signaling prevents inflammation and impacts composition of the microbiota during inflammation-induced colorectal cancer. Cancer Prev. Res. (Phila) 13, 25–40. 10.1158/1940-6207.Capr-19-0286 31771941

[B64] Ko J. S. Yang H. R. Chang J. Y. Seo J. K. (2007). *Lactobacillus Plantarum* inhibits epithelial barrier dysfunction and interleukin-8 secretion induced by tumor necrosis factor-α. World J. Gastroenterol. 13, 1962–1965. 10.3748/Wjg.V13.I13.1962 17461497 PMC4146973

[B65] Konishi H. Fujiya M. Tanaka H. Ueno N. Moriichi K. Sasajima J.. (2016). Probiotic-derived ferrichrome inhibits colon cancer progression via jnk-mediated apoptosis. Nat. Commun. 7, 12365–12377. 10.1038/Ncomms12365 27507542 PMC4987524

[B66] Kotzampassi K. Stavrou G. Damoraki G. Georgitsi M. Basdanis G. Tsaousi G.. (2015). A four-probiotics regimen reduces postoperative complications after colorectal surgery: a randomized, double-blind, placebo-controlled study. World J. Surg. 39, 2776–2783. 10.1007/S00268-015-3071-Z 25894405

[B67] Koundouros N. Poulogiannis G. (2018). Phosphoinositide 3-kinase/akt signaling and redox metabolism in cancer. Front. Oncol. 8, 160–169. 10.3389/Fonc.2018.00160 29868481 PMC5968394

[B68] Kuipers E. J. Grady W. M. Lieberman D. Seufferlein T. Sung J. J. Boelens P. G.. (2015). Colorectal cancer. Nat. Rev. Dis. Primers 1, 15065–15116. 10.1038/Nrdp.2015.65 27189416 PMC4874655

[B69] Kuugbee E. D. Shang X. Gamallat Y. Bamba D. Awadasseid A. Suliman M. A.. (2016). Structural change in microbiota by a probiotic cocktail enhances the gut barrier and reduces cancer via tlr2 signaling in a rat model of colon cancer. Dig. Dis. Sci. 61, 2908–2920. 10.1007/S10620-016-4238-7 27384052

[B70] Lai W. T. Huang F. C. (2019). Probiotics exert reciprocal effects on autophagy and interleukin-1β expression in *Salmonella*-infected intestinal epithelial cells via autophagy-related 16l1 protein. Benef. Microbes 10, 913–922. 10.3920/Bm2019.0046 31965835

[B71] Lan A. Lagadic-Gossmann D. Lemaire C. Brenner C. Jan G. (2007). Acidic extracellular ph shifts colorectal cancer cell death from apoptosis to necrosis upon exposure to propionate and acetate, major end-products of the human probiotic propionibacteria. Apoptosis 12, 573–591. 10.1007/S10495-006-0010-3 17195096

[B72] Lee J. Lee J.-E. Kim S. Kang D. Yoo H. M. (2020). Evaluating cell death using cell-free supernatant of probiotics in three-dimensional spheroid cultures of colorectal cancer cells. J. Vis. Exp. 160, E61285–61302. 10.3791/61285 32597876

[B73] Li S.-C. Hsu W.-F. Chang J.-S. Shih C.-K. (2019a). Combination of *lactobacillus acidophilus* and *bifidobacterium animalis* subsp. *lactis* shows a stronger anti-inflammatory effect than individual strains in Ht-29 cells. Nutrients 11, 969–986. 10.3390/Nu11050969 31035617 PMC6566532

[B74] Li S.-C. Lin H.-P. Chang J.-S. Shih C.-K. (2019b). *Lactobacillus Acidophilus*-fermented germinated brown rice suppresses preneoplastic lesions of the colon in rats. Nutrients 11, 2718–2734. 10.3390/Nu11112718 31717536 PMC6893647

[B75] Li Y.-P. Du X.-R. Zhang R. Yang Q. (2021). Interleukin-18 promotes the antitumor ability of natural killer cells in colorectal cancer via the Mir-574-3p/Tgf-β1 axis. Bioengineered 12, 763–778. 10.1080/21655979.2021.1880717 33660570 PMC8806203

[B76] Liu J. Huang X. E. (2015). Efficacy of bifidobacterium tetragenous viable bacteria tablets for cancer patients with functional constipation. Asian Pac. J. Cancer Prev. 15, 10241–10244. 10.7314/Apjcp.2014.15.23.10241 25556454

[B77] Liu Z.-H. Huang M.-J. Zhang X.-W. Wang L. Huang N.-Q. Peng H.. (2013). The Effects of perioperative probiotic treatment on serum zonulin concentration and subsequent postoperative infectious complications after colorectal cancer surgery: a double-center and double-blind randomized clinical trial. Am. J. Clin. Nutr. 97, 117–126. 10.3945/Ajcn.112.040949 23235200

[B78] Louis P. Hold G. L. Flint H. J. (2014). The gut microbiota, bacterial metabolites and colorectal cancer. Nat. Rev. Microbiol. 12, 661–672. 10.1038/Nrmicro3344 25198138

[B79] Ma E. L. Choi Y. J. Choi J. Pothoulakis C. Rhee S. H. Im E. (2010). The anticancer effect of probiotic *Bacillus Polyfermenticus* on human colon cancer cells is mediated through erbb2 and erbb3 inhibition. Int. J. Cancer 127, 780–790. 10.1002/Ijc.25011 19876926 PMC4420487

[B80] Madempudi R. S. Kalle A. M. (2017). Antiproliferative effects of *Bacillus coagulans* unique is2 in colon cancer cells. Nutr. Cancer 69, 1062–1068. 10.1080/01635581.2017.1359317 28910156

[B81] Mahkonen A. Putaala H. Mustonen H. Rautonen N. Puolakkainen P. (2008). *Lactobacillus Acidophilus* 74-2 and butyrate induce cyclooxygenase (cox)-1 expression in gastric cancer cells. Immunopharmacol. Immunotoxicol. 30, 503–518. 10.1080/08923970802135229 18618313

[B82] Mendes M. C. S. Paulino D. S. Brambilla S. R. Camargo J. A. Persinoti G. F. Carvalheira J. B. C. (2018). Microbiota modification by probiotic supplementation reduces colitis associated colon cancer in mice. World J. Gastroenterol. 24, 1995–2008. 10.3748/Wjg.V24.I18.1995 29760543 PMC5949713

[B83] Mi H. Dong Y. Zhang B. Wang H. Peter C. C. Gao P.. (2017). *Bifidobacterium Infantis* Ameliorates chemotherapy-induced intestinal mucositis via regulating t cell immunity in colorectal cancer rats. Cell Physiol. Biochem. 42, 2330–2341. 10.1159/000480005 28848081

[B84] Norouzi Z. Salimi A. Halabian R. Fahimi H. (2018). Nisin, a potent bacteriocin and anti-bacterial peptide, attenuates expression of metastatic genes in colorectal cancer cell lines. Microb. Pathog. 123, 183–189. 10.1016/J.Micpath.2018.07.006 30017942

[B85] Odamaki T. Sugahara H. Yonezawa S. Yaeshima T. Iwatsuki K. Tanabe S.. (2012). Effect of the oral intake of yogurt containing *Bifidobacterium Longum* Bb536 on the cell numbers of enterotoxigenic bacteroides fragilis in microbiota. Anaerobe 18, 14–18. 10.1016/J.Anaerobe.2011.11.004 22138361

[B86] Oh N. S. Joung J. Y. Lee J. Y. Kim Y. (2018). Probiotic and anti-inflammatory potential of *Lactobacillus Rhamnosus* 4b15 and lactobacillus gasseri 4m13 isolated from infant feces. Plos One 13, 1–15. 10.1371/Journal.Pone.0192021 29444150 PMC5812581

[B87] Oh N. S. Joung J. Y. Lee J. Y. Kim Y. J. Kim Y. Kim S. H. (2020). A synbiotic combination of *Lactobacillus Gasseri* 505 And *Cudrania Tricuspidata* leaf extract prevents hepatic toxicity induced by colorectal cancer in mice. J. Dairy Sci. 103, 2947–2955. 10.3168/Jds.2019-17411 32008775

[B88] O'keefe S. J. (2016). Diet, microorganisms and their metabolites, and colon cancer. Nat. Rev. Gastroenterol. Hepatol. 13, 691–706. 10.1038/Nrgastro.2016.165 27848961 PMC6312102

[B89] Osterlund P. Ruotsalainen T. Korpela R. Saxelin M. Ollus A. Valta P.. (2007). *Lactobacillus* supplementation for diarrhoea related to chemotherapy of colorectal cancer: a randomised study. Br. J. Cancer 97, 1028–1034. 10.1038/Sj.Bjc.6603990 17895895 PMC2360429

[B90] Parisa A. Roya G. Mahdi R. Shabnam R. Maryam E. Malihe T. (2020). Anti-cancer effects of *Bifidobacterium* species in colon cancer cells and a mouse model of carcinogenesis. Plos One 15, 1–18. 10.1371/Journal.Pone.0232930 32401801 PMC7219778

[B91] Pinzi L. Rastelli G. (2019). Molecular docking: shifting paradigms in drug discovery. Int. J. Mol. Sci. 20, 4331–4354. 10.3390/Ijms20184331 31487867 PMC6769923

[B92] Polakowski C. B. Kato M. Preti V. B. Schieferdecker M. E. M. Campos A. C. L. (2019). Impact of the preoperative use of synbiotics in colorectal cancer patients: a prospective, randomized, double-blind, placebo-controlled study. Nutrition 58, 40–46. 10.1016/J.Nut.2018.06.004 30278428

[B93] Priyadarshini S. Aich P. (2012). Effects of psychological stress on innate immunity and metabolism in humans: a systematic analysis. Plos One 7, 1–14. 10.1371/Journal.Pone.0043232 23028447 PMC3446986

[B94] Rafter J. Bennett M. Caderni G. Clune Y. Hughes R. Karlsson P. C.. (2007). Dietary synbiotics reduce cancer risk factors in polypectomized and colon cancer patients. Am. J. Clin. Nutr. 85, 488–496. 10.1093/Ajcn/85.2.488 17284748

[B95] Rawla P. Sunkara T. Barsouk A. (2019). Epidemiology of colorectal cancer: incidence, mortality, survival, and risk factors. Prz Gastroenterol. 2019;14:89–103. 10.5114/Pg.2018.81072 31616522 PMC6791134

[B96] Roller M. Clune Y. Collins K. Rechkemmer G. Watzl B. (2007). Consumption of prebiotic inulin enriched with oligofructose in combination with the probiotics *Lactobacillus rhamnosus* and *Bifidobacterium lactis* has minor effects on selected immune parameters in polypectomised and colon cancer patients. Br. J. Nutr. 97, 676–684. 10.1017/S0007114507450292 17349080

[B97] Rong J. Liu S. Hu C. Liu C. (2019). Single probiotic supplement suppresses colitis-associated colorectal tumorigenesis by modulating inflammatory development and microbial homeostasis. J. Gastroenterol. Hepatol. 34, 1182–1192. 10.1111/Jgh.14516 30357910

[B98] Saito Y. Hinoi T. Adachi T. Miguchi M. Niitsu H. Kochi M.. (2019). Synbiotics suppress colitis-induced tumorigenesis in a colon-specific cancer mouse model. Plos One 14, 1–19. 10.1371/Journal.Pone.0216393 31242213 PMC6594584

[B99] Saus E. Iraola-Guzman S. Willis J. R. Brunet-Vega A. Gabaldon T. (2019). Microbiome and colorectal cancer: roles in carcinogenesis and clinical potential. Mol. Aspects Med. 69, 93–106. 10.1016/J.Mam.2019.05.001 31082399 PMC6856719

[B100] Scartoni D. Desideri I. Giacomelli I. Di Cataldo V. Di Brina L. Mancuso A.. (2015). Nutritional supplement based on zinc, prebiotics, probiotics and vitamins to prevent radiation-related gastrointestinal disorders. Anticancer. Res. 35, 5687–5692. 26408744

[B101] Sentürk M. Ercan F. Yalcin S. (2020). The secondary metabolites produced by *Lactobacillus Plantarum* downregulate bcl-2 and buffy genes on breast cancer cell line and model organism drosophila melanogaster: molecular docking approach. Cancer Chemother. Pharmacol. 85, 33–45. 10.1007/S00280-019-03978-0 31673827

[B102] Sharifi M. Moridnia A. Mortazavi D. Salehi M. Bagheri M. Sheikhi A. (2017). Kefir: a powerful probiotics with anticancer properties. Med. Oncol. 34, 1–7. 10.1007/S12032-017-1044-9 28956261

[B103] Shokryazdan P. Sieo C. C. Kalavathy R. Liang J. B. Alitheen N. B. Faseleh Jahromi M.. (2014). Probiotic potential of *Lactobacillus* strains with antimicrobial activity against some human pathogenic strains. Biomed Res. Int. 2014, 1–17. 10.1155/2014/927268 25105147 PMC4106073

[B104] Song H. Wang W. Shen B. Jia H. Hou Z. Chen P.. (2018). Pretreatment with probiotic bifico ameliorates colitis-associated cancer in mice: transcriptome and gut flora profiling. Cancer Sci. 109, 666–677. 10.1111/Cas.13497 29288512 PMC5834773

[B105] Sung H. Ferlay J. Siegel R. L. Laversanne M. Soerjomataram I. Jemal A.. (2021). Global cancer statistics 2020: globocan estimates of incidence and mortality worldwide for 36 cancers in 185 countries. Ca Cancer J. Clin. 71, 209–249. 10.3322/Caac.21660 33538338

[B106] Talero E. Bolivar S. Ávila-Román J. Alcaide A. Fiorucci S. Motilva V. (2015). Inhibition of chronic ulcerative colitis-associated adenocarcinoma development in mice by vsl# 3. Inflamm. Bowel Dis. 21, 1027–1037. 10.1097/Mib.0000000000000346 25793324

[B107] Tiptiri-Kourpeti A. Spyridopoulou K. Santarmaki V. Aindelis G. Tompoulidou E. Lamprianidou E. E.. (2016). *Lactobacillus Casei* exerts anti-proliferative effects accompanied by apoptotic cell death and up-regulation of trail in colon carcinoma cells. Plos One 11, E0147960–E0147980. 10.1371/Journal.Pone.0147960 26849051 PMC4744000

[B108] Walia S. Kamal R. Dhawan D. Kanwar S. (2018). Chemoprevention by probiotics during 1, 2-dimethylhydrazine-induced colon carcinogenesis in rats. Dig. Dis. Sci. 63, 900–909. 10.1007/S10620-018-4949-Z 29427224

[B109] Walia S. Kamal R. Kanwar S. S. Dhawan D. K. (2015). Cyclooxygenase as a target in chemoprevention by probiotics during 1, 2-dimethylhydrazine induced colon carcinogenesis in rats. Nutr. Cancer 67, 603–611. 10.1080/01635581.2015.1011788 25811420

[B110] Wong S. H. Yu J. (2019). Gut microbiota in colorectal cancer: mechanisms of action and clinical applications. Nat. Rev. Gastroenterol. Hepatol. 16, 690–704. 10.1038/S41575-019-0209-8 31554963

[B111] Xu X. X. Bi J. P. Ping L. Li P. Li F. (2018). A network pharmacology approach to determine the synergetic mechanisms of herb couple for treating rheumatic arthritis. Drug Des. Devel. Ther. 12, 967–979. 10.2147/Dddt.S161904 29731604 PMC5923250

[B112] Yang S. C. Lin C. H. Sung C. T. Fang J. Y. (2014). Antibacterial activities of bacteriocins: application in foods and pharmaceuticals. Front Microbiol. 5, 241–251. 10.3389/Fmicb.2014.00241 24904554 PMC4033612

[B113] Yang T. Li X. Montazeri Z. Little J. Farrington S. M. Ioannidis J. P. A.. (2019). Gene-environment interactions and colorectal cancer risk: an umbrella review of systematic reviews and meta-analyses of observational studies. Int. J. Cancer 145, 2315–2329. 10.1002/Ijc.32057 30536881 PMC6767750

[B114] Yannai S. (2012). Dictionary Of Food Compounds With Cd-Rom. London: CRC Press.

[B115] Yue Y. Ye K. Lu J. Wang X. Zhang S. Liu L.. (2020). Probiotic strain *Lactobacillus Plantarum* yyc-3 prevents colon cancer in mice by regulating the tumour microenvironment. Biomed. Pharmacother. 127, 1–8. 10.1016/J.Biopha.2020.110159 32353824

[B116] Zaharuddin L. Mokhtar N. M. Muhammad Nawawi K. N. Raja Ali R. A. (2019). A randomized double-blind placebo-controlled trial of probiotics in post-surgical colorectal cancer. Bmc Gastroenterol. 19, 131–139. 10.1186/S12876-019-1047-4 31340751 PMC6657028

[B117] Zaylaa M. Alard J. Kassaa I. Peucelle V. Boutillier D. Desramaut J.. (2019). Autophagy: a novel mechanism involved in the anti-inflammatory abilities of probiotics. Cell Physiol. Biochem. 53, 774–793. 10.33594/000000172 31647207

[B118] Zhang J. W. Du P. Yang B. R. Gao J. Fang W. J. Ying C. M. (2012). Preoperative probiotics decrease postoperative infectious complications of colorectal cancer. Am. J. Med. Sci. 343, 199–205. 10.1097/Maj.0b013e31823aace6 22197980

[B119] Zhao S. Han J. Bie X. Lu Z. Zhang C. Lv F. (2016). Purification and characterization of plantaricin jla-9: a novel bacteriocin against *Bacillus* Spp. produced by *Lactobacillus Plantarum* Jla-9 from suan-tsai, a traditional chinese fermented cabbage. J. Agric. Food Chem. 64, 2754–2764. 10.1021/Acs.Jafc.5b05717 26985692

