# Supplementary material for: Effects of Probiotics at the Interface of Metabolism and Immunity to Prevent Colorectal Cancer-Associated Gut Inflammation: A Systematic Network and Meta-Analysis With Molecular Docking Studies
Source: Front Microbiol. 2022 May 27;13:878297. doi: 10.3389/fmicb.2022.878297 (PMC9195627; doi:10.3389/fmicb.2022.878297)
Supplement: Supplementary file 1 [file Data_Sheet_1.pdf]

Effects of Probiotics at the Interface of Metabolism and Immunity to Prevent Colorectal Cancer-associated Gut-inflammation: A Systematic Network and Meta-analysis with Molecular Docking Studies

Sinjini Patra[1], Nilanjan Sahu[2], Shivam Saxena[1], Biswaranjan Pradhan[3], Saroj Kumar Nayak[3] and Anasuya Roychowdhury[1#]

Affiliations: [1]Biochemistry and Cell Biology Laboratory, School of Basic Sciences, Indian Institute of Technology Bhubaneswar, Odisha, 752050, India; [2]National Institute of Science Education and Research (NISER) Bhubaneswar, HBNI, P.O. Bhimpur-Padanpur, Via: Jatni, Dist. Khurda, Odisha, 752050; [3]S. K. Dash Center of Excellence of Biosciences and Engineering & Technology (SKBET), Indian Institute of Technology Bhubaneswar, Odisha, 752050, India

Address for Correspondence: [#1]Biochemistry and Cell Biology Laboratory, School of Basic Sciences, Indian Institute of Technology Bhubaneswar, Odisha, 752050, India. Tel: +91-674-713-5106, e-mail: aroychowdhury@iitbbs.ac.in [ORCID ID: 0000-0003-3735-3021]

Materials and Methods

The data-mining strategy was used for automatic detection of the disease patterns from structured databases and finding out the functional genes that were comparatively unexplored. However, for more efficient screening, text-mining was further used. This novel information retrieval resource simultaneously constructed the interaction network from unstructured biomedical texts. Since the sole use of text-mining strategy might introduce biases towards dynamically studied disease phenotypes, to include the less explored studies, a unique combination of data- and text-mining techniques were implemented.

2.1. Meta-analysis establishes the rationale for probiotic intervention as an alternative strategy for the prevention and alleviation of CRC-related inflammation

In our meta-analysis study, the homogeneity of all effect sizes in the sample of individual clinical trials (from the same population) was assessed by Q-statistics. The degree of heterogeneity was indicated by the I^2 index. The efficacy of probiotic application depended on age group, specific strains, mode of administration, and clinical dosages. Randomized, double-blind placebo-controlled human clinical trials were assessed. Standardized mean difference (SMD) or mean difference (MD), 95% confidence interval (CI), effect size, and weight percentage of studies were used to estimate the overall effects of specific probiotic strains by using a fixed-effects model. The standard deviation, *i.e.*, the mean change in the number of patients and healthy individuals, was used to estimate the mean difference between the placebo and the probiotics or probiotic formulation (synbiotics) groups. Estimated effects were calculated using RevMan 5.4.1 software with a Mantel–Haenszel fixed-effects model. Heterogeneity was indicated by the Q statistic p-value < 0.05, and I^2 values higher than 40% denoted the heterogeneity of the data. All the estimated p-values were two-sided, and statistical significance for all included studies were considered for p-values ≤ 0.05.

2.2. Data sources and search strategy using the data-mining approach

The data-mining or manual analysis was performed reference management software. The search terms or MeSH terms used in EndNote included “probiotics” ∩ “colon cancer,” “probiotics” ∩ “colorectal cancer,” “probiotics” ∩ “bowel cancer,” “probiotics” ∩ “rectal cancer,” “probiotics” ∩ “colon adenocarcinoma.” The search for the present study was restricted to the literature available from the year 2000 to June 2021 and the work conducted on humans.

2.3. Selection of the genes and network construction using the text-mining approach

Cytoscape helps to envisage the interaction networks incorporating the expression profiles of the corresponding training genes. A meta search tool, Agilent Literature Search Software, was used in conjunction with Cytoscape to recognize the association network involving the training genes of interest. Agilent Literature Search Software automatically collected the retrieved documents from their respective sources whenever a query was entered and submitted to different text-based search engines. The raised documents were then parsed into sentences. The gene names

in the sentences were then defined as “concepts,” whereas “verbs” denoted the interaction terms of interests using a lexicon set. The network was developed by eliciting an association for every parsed sentence containing one “verb” or known interaction term and two “concepts.” The gene names are represented as nodes and association terms designated as edges in the text-mining-based network, generated through Cytoscape. In Cytoscape, “Search terms” represented the training genes; Max Engine Matches was fixed at 10; “Use Context,” and the “Concept Lexicon Restrict Search” were recognized as search controls; and “Extraction control” was set as “*Homo sapiens*.”

2.4. Data extraction using Molecular Complex Detection analysis

Molecular Complex Detection (MCODE) plug-in of Cytoscape was applied to weigh nodes by local neighborhood density and graphically display the extracted module as an isolated, more significant compact region. The live Network view of the clusters aided in understanding the functional features and local topology in the whole network, containing other candidate genes and edges. MCODE tool made groups of “genes of interest” according to the similarity of their biological functions defined by correlation coefficient and considered an edge in the network according to its highest score among all the candidate edges for the respective genes. This method, called the “top overlap” method, was preferable as it had a directed mode that consents to fine-tuning related cluster interconnectivity amid the rest of the network. This increased the accuracy and robustness of the analysis, minimizing the likelihood of false-positive results.

2.5. Quantitative data synthesis by Network Analysis

The clustering algorithm of the device determined the clustering coefficient and node degree of the respective node genes. These implied the degree of involvement for a node in the participating clusters and the number of connections linked to an individual node. The network thus generated visually represented the node degree with functional relationship and had only undirected edges indicating the mutual associations of the genes. Quantitative data analysis of the association network also presented the involvement of individual candidate genes in the respective clusters, signifying their functional annotation. This tool could not extract the specific biological processes, like metabolic pathways, apoptotic responses, and immunomodulatory outcomes. The process continued with further analysis of the genes with the highest MCODE score of pathways associated with them.

2.6. Assessment of gene ontology overrepresentation: The Biological Networks Gene Ontology (BiNGO) analysis

In the BiNGO tool, a threshold value of $p<0.001$ was set to find out statistically overrepresented GO terms for a gene set associated with biological processes according to the GO database. The MCODE-derived gene clusters of the association network were subjected to the BiNGO analysis. Statistical test was fixed to “hypergeometric test,” multiple testing correction as “Benjamini & Hochberg False Discovery Rate (FDR) correction,” 0.05 was considered as the significance level, the categories to be visualized were “overrepresented categories after correction,” reference set was “use the whole annotation as the reference set,” ontology file was “GO_Biological_Process.” Next, “*Homo sapiens*” was chosen as organism/annotation.

2.7. Identification of significant candidate gene-associated enriched pathways involved with CRC associated inflammation

This tool performs integrative analysis of human gene sets. It can also find functional associations between known cellular pathways and processes and corresponding genes using topologic analysis and protein-interaction networks. The association network was applied for "enrichment analysis," molecular network parameter was set at "STRING," identifier format "HGNC SYMBOL," reference database was selected as "KEGG."

3. Results

3.3. Generation of candidate gene-association network of probiotic-inflammation-CRC axis

In the text-mining-based network, the respective candidate genes indicated a more common type of associations for which structured network interaction data was limited. According to the parameter statistics, the network connectivity was represented by the "number of connected components," which indicated a pairwise connection among all the nodes. Therefore, a lesser number of connected components of 30 suggested strong connectivity of the association network (**Table 3**). The clustering coefficient 0.559 proposed the degree of involvement of individual nodes in the corresponding network cluster, with a 5.270 average number of neighbors. The absence of isolated nodes and high "network heterogeneity" of 1.269 defined the inclination to form hub nodes or high degree nodes. The involvement of multiple biologically relevant pathways with the real functional network was indicated by the presence of hub nodes.

3.5. Annotation profile of the sub-clusters signified a functional contribution of probiotics in the prevention of chronic inflammation related to CRC

In the MCODE analysis, the module with the highest MCODE score should have possessed the most relevant genes involved in the bioprotective functions of probiotics against oncogenic gut-inflammation. Additionally, the network topologies like the clustering coefficient and node degree designated the degree of involvement of the nodes in the specific module. A higher node degree reflected a multi-functional role of the corresponding gene, which is interconnected with more than one associative gene. Therefore, individual genes with a high MCODE score, clustering coefficient, and node degree could be considered as the principal regulatory gene of the disease network. At the same time, the associated module could be assigned to have a potential role in the functional profiles of the respective nodes.

Supplementary figures and figure legends

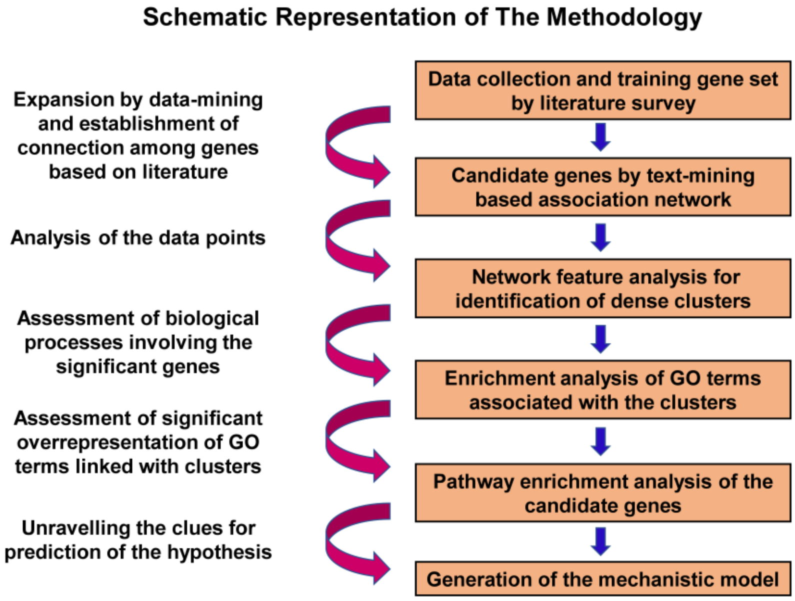

Supplementary Figure 1 The schematic representation of the methodologies for the systematic network analysis on probiotic-mediated prevention of CRC-pathogenesis related gut-inflammation.

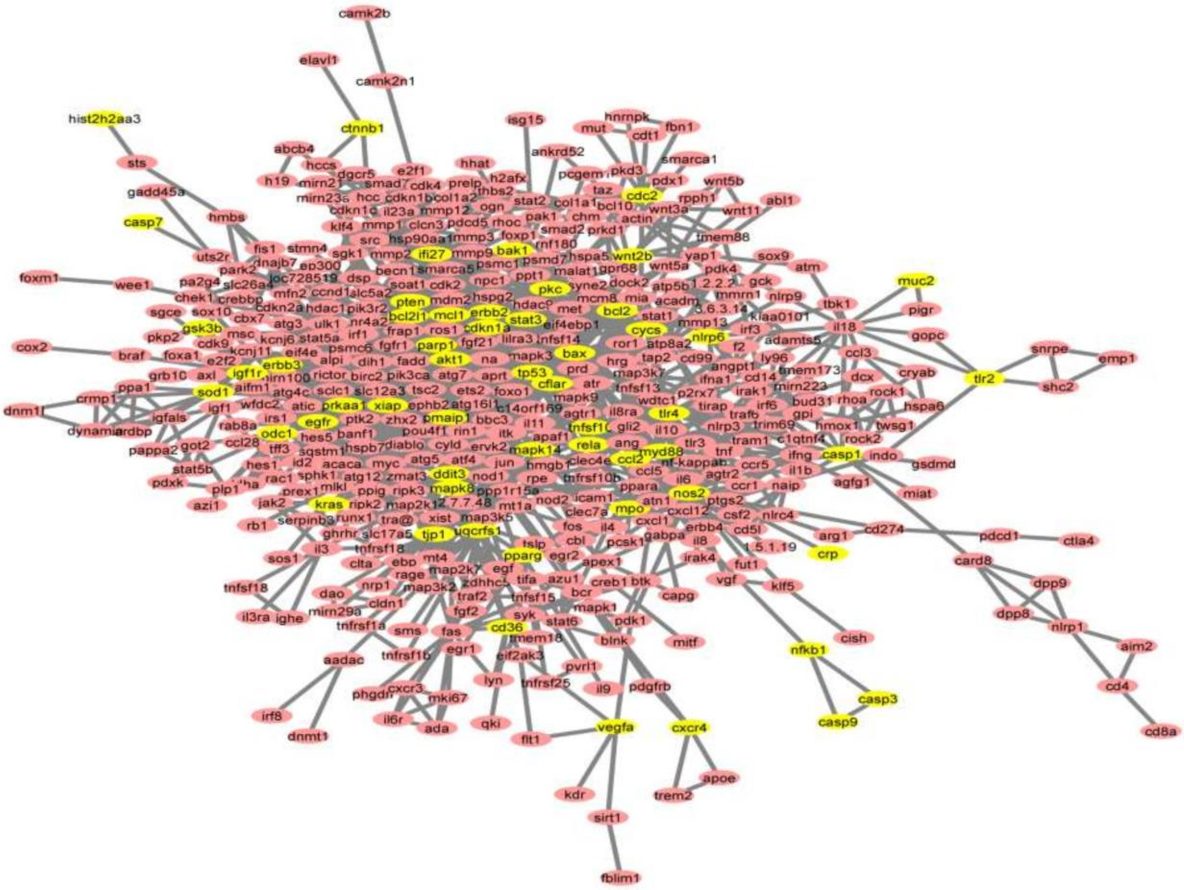

Supplementary Figure 2 Association network topologies for probiotic intervention in colorectal carcinogenesis. The nodes of the association network (red and yellow) represent the candidate genes associated with probiotic administration in CRC pathogenesis. The training genes for probiotic-CRC axis are represented in yellow. The nodes of the association network are interconnected by edges (grey). The edges represent the multifunctional nature of the candidate genes (nodes) and the number of literatures supporting the relationship between the nodes.

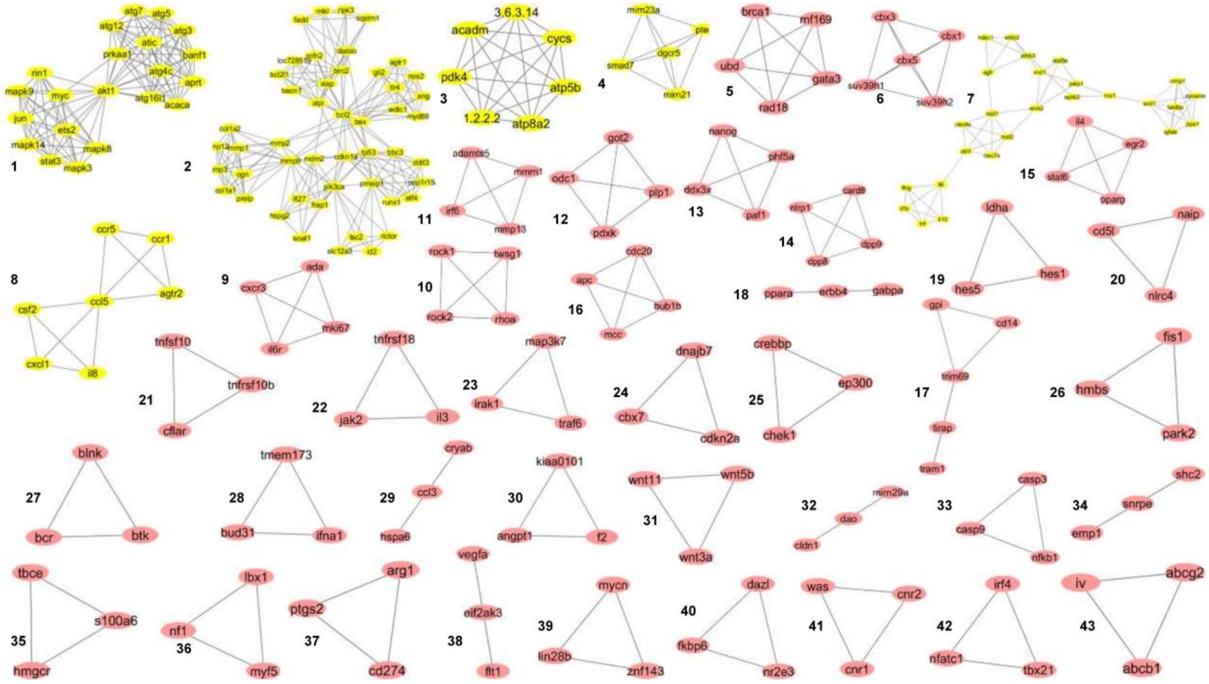

Supplementary Figure 3 Network topologies of MCODE clusters derived from the association network for probiotic application in CRC pathogenesis-related gut-inflammation. Network topologies with a minimum MCODE score of 3 are depicted out of all networks (43). The significant clusters (yellow, with a minimum score of 4 with 7 nodes and 12 edges) are identified for further pathway analysis. The selected MCODE clusters (yellow) represents highly interconnected nodes among the whole network with significant genes or associated pathways involved in the disease pathogenesis.

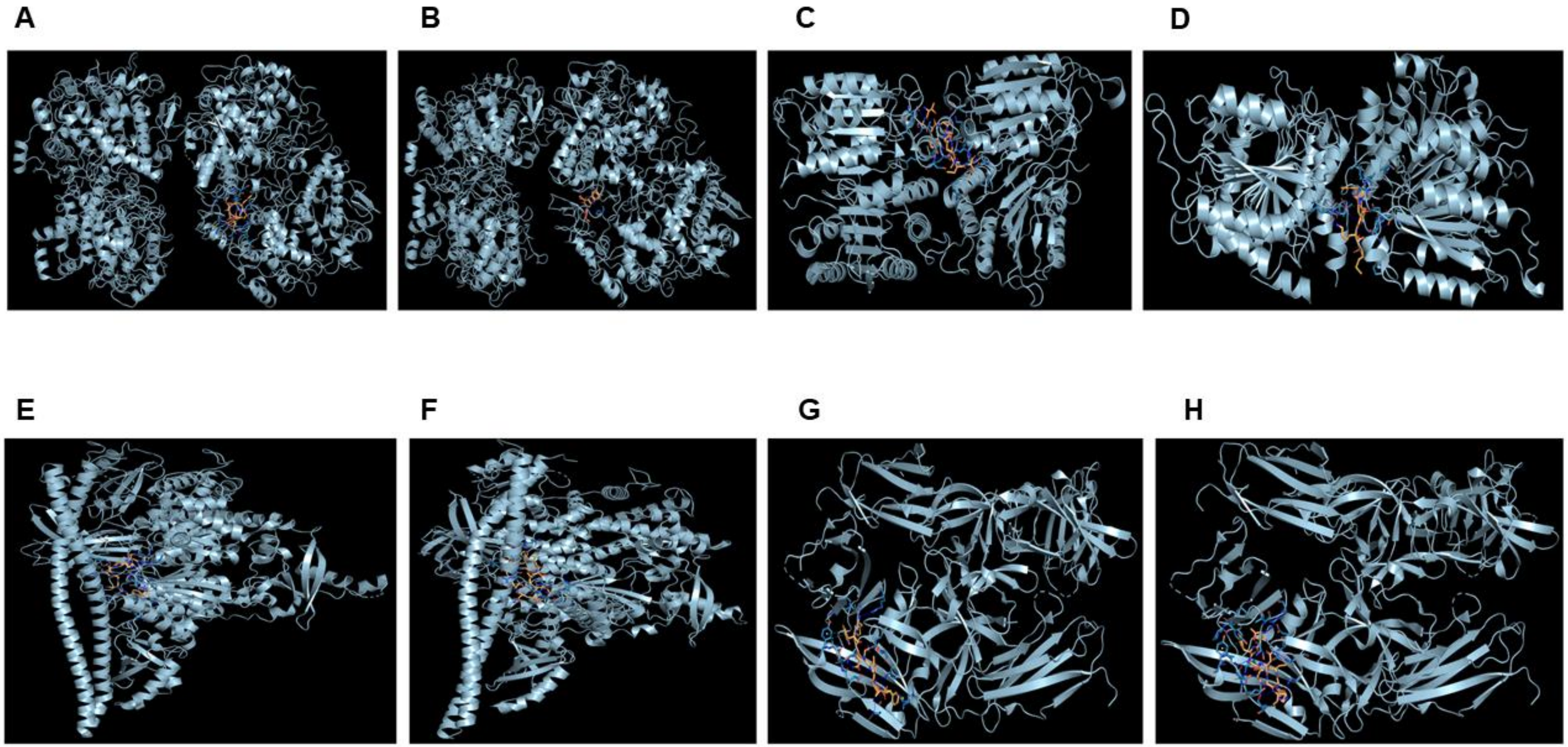

Supplementary Figure 4 Molecular docking studies of CRC-associated target proteins with probiotic-derived bacteriocins. COX-2 with Plantaricin JLA-9 (A). COX-2 with lactococcin A (B). CASP9 with Lactococcin mmfii (C). CASP9 with Plantaricin JLA-9 (D). PI3K with Lactococcin mmfii (E). PI3K with Plantaricin W (F). IL18R with Plantaricin JLA-9 (G). IL18R with Lactococcin mmfii (H).

Supplementary tables

*** Supplementary Table 1 The association network statistics of probiotic-CRC axis**

	Topology feature details						
**Network**	**Network heterogeneity**	**Number of Nodes**	**Nodes Edges**	**No. of isolated nodes**	**Avg. num. of neighbours**	**Clustering coefficient**	**Number of connected components**
Training gene set	1.269	540	1423	0	5.270	0.559	30

* The table contains details of the primary association network obtained by text-mining results using an Agilent Literature search (ALS) plugin. "Network clustering coefficient' is the average of the clustering coefficients for all nodes in the network, and the 'average number of neighbours' indicates the average connectivity of a node in the network.

Supplementary Table 2 MCODE score, number of nodes and edges of clusters from association network for probiotic application in CRC-associated gut-inflammation

MCODE cluster	Score	Number of nodes	Number of edges
*1	11.2	21	112
*2	8.894	48	209
*3	7	7	21
*4	5	5	10
5	5	5	10
6	5	5	10
*7	4.480	26	56
*8	4	7	12
9	4	4	6
10	4	4	6
11	4	4	6
12	4	4	6
13	4	4	6
14	4	4	6
15	4	4	6
16	4	4	6
17	3	5	6
18	3	3	3
19	3	3	3
20	3	3	3
21	3	3	3
22	3	3	3
23	3	3	3
24	3	3	3
25	3	3	3
26	3	3	3
27	3	3	3
28	3	3	3
29	3	3	3
30	3	3	3
31	3	3	3
32	3	3	3
33	3	3	3
34	3	3	3
35	3	3	3
36	3	3	3
37	3	3	3
38	3	3	3
39	3	3	3
40	3	3	3
41	3	3	3
42	3	3	3
43	3	3	3

* The modules which are selected for pathway analysis

Supplementary Table 3 GO enrichment analysis by BiNGO of the MCODE clusters

MCODE derived cluster	BINGO derived overrepresented GO terms
Cluster 1	Autophagic vacuole assembly, cellular response to stress, extracellular stimulus, nutrient levels, positive regulation of metabolic processes, intracellular protein kinase cascade, induction of apoptosis, signal transmission via phosphorylation, stress activated protein kinase cascade (JNK, MAPKKK)
Cluster 2	Cellular response to stress, external stimulus, organic substances, Positive regulation of cellular biological processes, apoptosis, release of cytochrome c, apoptotic mitochondrial changes, regulation of cell proliferation, positive regulation of hydrolase and peptidase activity, activation of caspases, response to oxygen levels, collagen metabolic processes, multicellular catabolic processes, response to endoplasmic reticulum stress, regulation of mitochondrial membrane potential and permeability, regulation of interleukin 17 and 23 production, intracellular protein kinase and metabolic processes, regulation of nf-κb signaling pathway, positive regulation of phospholipase A2 and phospholipase C activity
Cluster 3	Cellular nitrogen compound biosynthetic process, ATP biosynthetic and metabolic process, purine ribonucleoside triphosphate biosynthetic process, medium-chain fatty acid metabolic process, fatty acid beta-oxidation using acyl-CoA dehydrogenase
Cluster 4	Regulation of cell-cell adhesion, cell-matrix adhesion, epithelial to mesenchymal transition, regulation of protein modification, phosphate metabolic processes, cell migration, adherent junction organization, cellular protein localization, protein amino acid phosphorylation, regulation of cell differentiation and development

Cluster 7	Positive regulation of cytokine (IL6) and chemokine production, apoptosis, T cell, lymphocyte, leukocyte activation and proliferation, regulation of lymphocyte and leukocyte mediated immunity, inflammatory responses, cellular defense processes, adaptive immunity, leukocyte migration, cellular response to stress and stimulus, bacterium, biotic stimulus or organic substances, intracellular protein kinase cascades (JNK, MAPKKK, ERK1, ERK2), regulation of nitric oxide biosynthetic processes, regulation of monooxygenase and oxidoreductase activity
Cluster 8	Immune response, locomotion, chemotaxis, G-protein coupled receptor signaling pathway, jak-stat pathway, inflammatory processes, response to stress, cellular metal ion homeostasis

Supplementary Table 4 32 significant pathways found by JEPETTO

Serial No.	Pathway	XD-score	q-Value	Overlap/size
1.	Bladder cancer	2.33020	0.00000	21/38
2.	Colorectal cancer	1.63831	0.00000	26/61
3.	Pancreatic cancer	1.62132	0.00000	29/70
4.	Chronic myeloid leukemia	1.61356	0.00000	29/69
5.	NOD-like receptor signaling pathway	1.60115	0.00000	28/59
6.	Prostate cancer	1.50998	0.00000	38/84
7.	Toll-like receptor signaling pathway	1.45953	0.00000	37/90
8.	Glioma	1.43925	0.00000	26/60
9.	Regulation of autophagy	1.41186	0.00009	9/21
10.	Endometrial cancer	1.39126	0.00000	20/50
11.	Leishmaniasis	1.30498	0.00000	22/62
12.	Amyotrophic lateral sclerosis (ALS)	1.28372	0.00000	16/47
13.	Melanoma	1.20516	0.00000	25/62
14.	Apoptosis	1.19780	0.00000	31/81
15.	Non-small cell lung cancer	1.19498	0.00000	18/51
16.	ErbB signaling pathway	1.14447	0.00000	32/84
17.	Shigellosis	1.13275	0.00000	18/56
18.	Dorso-ventral axis formation	1.12859	0.00273	7/20
19.	Malaria	1.09969	0.00000	16/42
20.	p53 signaling pathway	1.07401	0.00000	23/62
21.	Small cell lung cancer	1.07122	0.00000	27/82
22.	Chagas disease	0.94965	0.00000	32/99
23.	Acute myeloid leukemia	0.89855	0.00001	16/52
24.	Epithelial cell signaling in Helicobacter pylori infection	0.84252	0.00002	17/59
25.	RIG-I-like receptor signaling pathway	0.80938	0.00000	18/55
26.	Thyroid cancer	0.80405	0.00232	8/25
27.	T cell receptor signaling pathway	0.77736	0.00000	32/102
28.	VEGF signaling pathway	0.75206	0.00001	18/62
29.	Fc epsilon RI signaling pathway	0.70925	0.00000	19/65
30.	B cell receptor signaling pathway	0.69314	0.00001	19/69
31.	Neurotrophin signaling pathway	0.69064	0.00000	33/121
32.	Pathways in cancer	0.62231	0.00000	84/304

Cluster 1

1.	Regulation of autophagy	1.12068	0.00000	5/21
2.	RIG-I-like receptor signaling pathway	0.38475	0.00008	5/55
3.	Shigellosis	0.37663	0.00008	5/56
4.	One carbon pool by folate	0.33020	0.22414	1/10
5.	Dorso-ventral axis formation	0.33020	0.02053	2/20

6.	Colorectal cancer	0.32364	0.00001	6/61
7.	Adipocytokine signaling pathway	0.28108	0.00008	5/57
8.	Fc epsilon RI signaling pathway	0.23789	0.00014	5/65
9.	ErbB signaling pathway	0.21592	0.00006	6/84
10.	Pancreatic cancer	0.21592	0.00015	5/70
**Cluster 2**				
11.	Bladder cancer	0.55626	0.00003	6/38
12.	Apoptosis	0.49258	0.00000	9/81
13.	Amyotrophic lateral sclerosis (ALS)	0.47787	0.01484	4/47
14.	Small cell lung cancer	0.38809	0.00017	7/82
15.	p53 signaling pathway	0.37629	0.00003	7/62
16.	Chronic myeloid leukemia	0.34497	0.00067	6/69
17.	Prostate cancer	0.26992	0.00179	6/84
18.	Colorectal cancer	0.26894	0.02603	4/61
**Cluster 7**				
19.	Graft-versus-host disease	0.43944	0.00660	3/25
20.	Malaria	0.34039	0.00297	4/42
21.	Prion diseases	0.30230	0.01100	3/35
22.	Allograft rejection	0.27944	0.06791	2/25
23.	Type I diabetes mellitus	0.23530	0.07706	2/29
24.	NOD-like receptor signaling pathway	0.23063	0.00579	4/59
25.	Intestinal immune network for IgA production	0.18166	0.10925	2/36

Supplementary Table 5 PDB IDs of the proteins and PubChem IDs of the probiotic-derived bacteriocins

Protein	PDB ID	Method	Resolution	Ligand	PubChem ID	Property
Cox2	1CX2	X-RAY DIFFRACTION	3.00 Å	Plantaricin JLA-9	132535900	Antibacterial (Zhao et al., 2016)
						Antiviral (Anwar et al., 2020)
Caspase9	2AR9	X-RAY DIFFRACTION	2.80 Å	Lactococcin A	371544	Antibacterial (Holo et al., 1991)
PI3K	4WAF	X-RAY DIFFRACTION	2.39 Å	Lactococcin mmfii	139588229	Antibacterial (Ferchichi et al., 2001)
IL18R	4R6U	X-RAY DIFFRACTION	2.80 Å	Plantaricin W	139586573	Antibacterial (Holo et al., 2001)
AKT	4GV1	X-RAY DIFFRACTION	1.49 Å	Bacteriocin 28b	387237	Antibacterial (Yang et al., 2014)
TLR4	3VQ2	X-RAY DIFFRACTION	2.48 Å	Plantaricin D	139586697	Antibacterial (Aymerich et al., 2000)
IκB-α	1IKN	X-RAY DIFFRACTION	2.30 Å	Plantaricin BN	380907	Antibacterial (Yannai, 2012)
						Antiviral (Anwar et al., 2020)
p38 alpha	1OVE	X-RAY DIFFRACTION	2.10 Å			

mTOR	4DRH	X-RAY DIFFRACTION	2.30 Å
Caspase-3	1NMS	X-RAY DIFFRACTION	1.70 Å
Cytochrome c	3NWV	X-RAY DIFFRACTION	1.90 Å
ATG4	2Z0D	X-RAY DIFFRACTION	1.90 Å

Supplementary Table 6 Binding free energy from molecular docking analysis based on interaction of seven different bacteriocins with CRC associated target proteins

	Binding Energy (Kcal/mol)

	Lactococcin mmfii	Plantaricin BN	Plantaricin JLA-9	Plantaricin W	Plantaricin D	Lactococcin A	Bacteriocin 28b
**BCL-2**	-5.5	-4.9	-5.2	-5.1	-5.4	-7	-6.4
**Cytochrome C**	-7.4	-6.3	-7.8	-7.2	-6.3	-7	-7.1
**Caspase 9**	-9.2	-6.5	-8.8	-7.5	-7.9	-7.6	-8.2
**Caspase 3**	-7.1	-6.5	-7.8	-6.1	-6.8	-8.3	-8.1
**PI3K**	-9	-7.5	-7.6	-8.5	-7.7	-7.9	-7.9
**AKT**	-7.6	-7.6	-8.7	-6.7	-7.5	-7.6	-8.3
**mTOR**	-7.9	-6.9	-7.1	-7.1	-7	-7.4	-7.8
**ATG4**	-6.4	-5.8	-6.1	-5	-6	-7.6	-7.8
**STAT3**	-6.8	-6.2	-7.3	-6.5	-5.5	-7.8	-6.9
**p38 alpha**	-6.9	-6.6	-7.2	-6.7	-6.5	-8.1	-7.3
**NF-Kb**	-6.1	-5.5	-7.8	-5.2	-5.8	-7.6	-8
**BCL2-XL**	-7.3	-6.6	-8.2	-7.2	-6.7	-7.4	-6.8
**BCL2**	-6.6	-6.2	-6	-6.1	-6.9	-8.3	-7.8
**TLR4**	-7.9	-7.2	-7.8	-7.8	-7.2	-7.8	-7.4
**CXCL8**	-6	-4.7	-5.7	-4.6	-5.1	-6.1	-6.6
**IκB-α**	-7.4	-7.4	-8.1	-6.9	-6.4	-8.4	-7.8
**BAX**	-6.4	-5.7	-7	-4.5	-6	-8.3	-8.4
**COX2**	-8.8	-7	-11	-8.6	-8.6	-9.6	-9.1
**TLR4**	-7.6	-6.5	-7.6	-7.1	-7.8	-7.6	-8
**EGFR**	-6	-6	-6.2	-5.4	-4.9	-6.2	-7.3
**IL18**	-8.3	-7.3	-8.4	-6.8	-7.6	-7.8	-7.5
**IL6**	-6.2	-6.3	-6.9	-5.7	-5	-7.3	-6.6
**TNFR**	-6.5	-5.7	-6.9	-6.2	-6.1	-8.3	-7.3

Supplementary Table 7 Interaction of bacteriocins with AKT, TLR4, IκB-α, p38 alpha, mTOR, CASP-3, CYTOCHROME C, ATG4 by molecular docking

Ligands	Proteins							
	AKT (4GV1)	TLR4 (3VQ2)	IκB-α (1IKN)	p38 alpha (1OVE)	mTOR (4DRH)	Caspase-3 (1NMS)	Cytochrome c (3NWV)	ATG4 (2Z0D)
**Plantaricin JLA-9**	**Hydrogen bond:** THR160, ARG243, GLU314, TYR315, GLU341, TYR350<br>**Hydrophobic Interaction:** PHE161, LEU239, ASP292, LEU295, THR312, TYR315, ARG346, TYR350	**Hydrogen bond:** GLU92<br>**Hydrophobic Interaction:** ARG337, PHE406, ILE411<br>**pi-Cation Interaction:** ARG434	**Hydrogen bond:** PRO27, LYS28, GLN29, ARG33, SER51, THR57, GLU222, GLU225, ARG236, GLN241, ARG260<br>**Hydrophobic Interaction:** LYS28, THR52, ASP53, LYS56, GLU222, PHE239, PRO261, PRO275<br>**Salt Bridge:** HIS58	**Hydrogen bond:** SER37, LYS53, SER56, ARG67, GLU71, ASP150, LYS152, ASN155, ASP168, ARG173, HIS174, GLU178<br>**Hydrophobic Interaction:** ASP168, LEU171, ARG173, GLU178, VAL183, THR185	**Hydrogen bond:** GLY84, LYS88, PRO109, ALA112, TYR113, SER115, ALA116, GLU2022, PHE2039, ARG2042, THR2064, LYS2066<br>**Hydrophobic Interaction:** GLU110, TYR113, ALA116, PRO2021, GLU2022, THR2064	**Hydrogen bond:** GLU124, GLY125, LEU136, LYS137, ARG164, TYR197, PRO201<br>**Hydrophobic Interaction:** LEU136, LYS137, GLU167, TYR195, PRO201, TYR203	**Hydrogen bond:** GLN42, GLY56, GLU61, ASP62, GLU66<br>**Hydrophobic Interaction:** ILE57, ILE58, GLU61, ASP62, MET65, GLU66, ALA92, ILE95<br>**Salt Bridge:** LYS88	**Hydrogen bond:** GLY40, GLN43, GLU117, THR118, GLN141, TRP142, ASN261<br>**Hydrophobic Interaction:** GLU117, TRP142, THR233
**Lactococcin A**	**Hydrogen bond:** GLU191, LYS276, ASN279<br>**Hydrophobic Interaction:** LEU295<br>**Salt Bridge:** LYS276	**Hydrogen bond:** ASN456, GLN505, ASN529<br>**Hydrophobic Interaction:** TYR454, LYS503, PHE551, ARG553	**Hydrogen bond:** THR179, GLN212<br>**Hydrophobic Interaction:** TYR181, PRO344, LEU346	**Hydrogen bond:** ARG23, ASP88<br>**Hydrophobic Interaction:** ARG5, PRO6, ARG23, LYS45	**Hydrogen bond:** GLU2022, ARG2036, GLY2040<br>**Hydrophobic Interaction:** TYR113	**Hydrogen bond:** ASP179, ASP180, ASN208<br>**Hydrophobic Interaction:** ALA183, PHE250	**Hydrogen bond:** ASN103<br>**Hydrophobic Interaction:** LYS99, LYS100, GLU104	**Hydrogen bond:** LEU43, GLN100<br>**Hydrophobic Interaction:** PHE16, LEU43
**Lactococcin**	**Hydrogen bond:**	**Hydrogen bond:**	**Hydrogen bond:**	**Hydrogen bond:**	**Hydrogen bond:**	**Hydrogen bond:**	**Hydrogen bond:**	**Hydrogen bond:**

**mmfii**	THR1, ASP274, LYS276, GLY294, GLU314, TYR315, TYR350, GLN352<br>**Hydrophobic Interaction:** PHE236, LEU295, THR312, GLU314, TYR315, LEU347, TYR350	THR1, GLU92, VAL93, LYS263, ARG337, ARG434<br>**Hydrophobic Interaction:** ARG337, MET358	THR1, ARG143, ASN182, ASN200, ARG201, SER205, GLY209, ASP210, GLU211, ARG253, ASP294, ARG295<br>**Hydrophobic Interaction:** THR1, PHE142, ARG143, ILE192	THR1, ALA34, SER37, LYS53, SER56, GLU71, ASP150, LYS152, ASN155, ASP168, LEU171, ARG173, HIS174<br>**Hydrophobic Interaction:** ALA34, ARG67, GLU178, THR185	THR1, VAL86, LYS88, GLU2041, ARG2042, ASN2043, ALA2056, GLU2067, TYR2088<br>**Hydrophobic Interaction:** ILE87, LEU119, GLU2041	GLU124, GLY125, LEU136, LYS137, THR166, GLU167, TYR203<br>**Hydrophobic Interaction:** ILE126, LYS137, GLU167, TYR203<br>**pi-Cation:** LYS137	GLY1, THR1, GLY56, GLU61, ASP62, GLU69, LYS88, ILE95<br>**Hydrophobic Interaction:** ASP62, THR63, LYS88, GLU89, ALA92, ILE95	THR1, GLY40, GLU117, THR118, GLN141, TRP142, ARG229, ASN261<br>**Hydrophobic Interaction:** GLU117, TRP142<br>**pi-Stacking:** TRP142
**Plantaricin W**	**Hydrogen bond:** THR160, ASP190, GLU191, ARG243, LYS276, GLU278, ASP292, GLY294, LEU295, GLY311, GLU341<br>**Hydrophobic Interaction:** PHE161, LEU295, TYR315, ARG346, TYR350<br>**Salt Bridge:** ASP292	**Hydrogen bond:** ARG90, LYS91, GLU92, ARG337, ARG380, ALA382, HIS429, LYS433, ARG434, ASN456, LYS458, GLY478, LYS503, GLN505<br>**Hydrophobic Interaction:** MET358, LYS360, ALA382, PHE406, ILE411, THR436<br>**Salt Bridge:** GLU437<br>**pi-Cation:** LYS263	**Hydrogen bond:** ARG143, GLU153, ASN182, HIS184, ASN200, ARG201, SER205, ASP210, ARG253, TYR267, THR292, ASP294<br>**Hydrophobic Interaction:** PHE142, ILE192, LEU207, GLU211, LEU227, LEU289, ASP293<br>**Salt Bridge:** GLU211, GLU213, ASP254, GLU265	**Hydrogen bond:** THR16, GLY36, SER37, LYS54, SER56, HIS64, ARG67, THR68, LYS152, SER154, ASN155, ASP168, ARG173, THR175, ALA184, THR221<br>**Hydrophobic Interaction:** TYR35, ARG67, ASP168, LEU171, THR185<br>**Salt Bridge:** ASP150, ASP168<br>**pi-Stacking:** TRP187	**Hydrogen bond:** LYS88, SER115, ALA116, ASP2020, GLU2022, GLU2041, ARG2042, MET2057, PRO2062, GLN2063, TYR2088<br>**Hydrophobic Interaction:** GLU2022, VAL2044, ALA2056, ARG2060, TYR2088, VAL2094<br>**Salt Bridge:** GLU2067	**Hydrogen bond:** GLU124, LEU136, LYS137, ARG164, GLU167, TYR197<br>**Hydrophobic Interaction:** GLU124, LEU136, LYS137, GLU167, TYR195, PRO201<br>**Salt Bridge:** GLU123, GLU167	**Hydrogen bond:** GLY1, ARG38, LYS39, ASN54, GLU61, ASP62<br>**Hydrophobic Interaction:** GLU61, ASP62, LYS88, GLU89, ALA92, ILE95, ALA96, LYS99<br>**Salt Bridge:** GLU61, ASP62	**Hydrogen bond:** SER61, LYS65, LEU73, ASN74, ALA75, HIS167, MET170, GLU178, GLU350<br>**Hydrophobic Interaction:** LYS65, MET170, THR173, GLU178, ARG181, LEU182, VAL352<br>**Salt Bridge:** GLU178

**Bacteriocin 28b**	**Hydrogen bond:** ASP274, ASP292, GLY294, LEU295 **Hydrophobic Interaction:** PHE161	**Hydrogen bond:** GLN505, ASN529 **Hydrophobic Interaction:** LYS503, GLN505	**Hydrogen bond:** GLY183, THR256, SER326 **Hydrophobic Interaction:** TYR181, LEU346	**Hydrogen bond:** THR91, ALA93 **Hydrophobic Interaction:** PRO6, PHE8, PRO21, PHE90	**Hydrogen bond:** HIS71, VAL78, ASP2102, ARG2109 **Hydrophobic Interaction:** GLU75, ASP2102, LEU2103	**Hydrogen bond:** ASP180, GLU248, PHE250 **Hydrophobic Interaction:** GLU246, PHE247 **pi-Stacking:** PHE247	**Hydrogen bond:** MET65, GLU66, GLU69, ARG91 **Hydrophobic Interaction:** GLU66, LYS88	**Hydrogen bond:** PHE13, HIS303 **Hydrophobic Interaction:** TRP98, GLN302
**Plantaricin D**	**Hydrogen bond:** LYS158, ARG243, ASP274, LYS276, GLU278, ASP292, GLY294, LEU295, TYR315, GLU341, GLY345 **Hydrophobic Interaction:** THR160, PHE161, LEU239, ASP292, ARG346, TYR350	**Hydrogen bond:** ARG90, LYS360, PHE406, SER413, HIS429, LYS433, ARG434, TYR454 **Hydrophobic Interaction:** LYS360, ILE411, TYR454	**Hydrogen bond:** PHE142, ASN182, HIS193, ARG201, ASP210, GLU211, ARG253, ARG255 **Hydrophobic Interaction:** ARG143, LEU189, ARG255	**Hydrogen bond:** ALA34, SER37, LYS54, SER56, GLN60, HIS64, GLU71, ARG173, GLU178 **Hydrophobic Interaction:** THR16, ALA34, LYS53, GLU178, THR185	**Hydrogen bond:** LYS88, GLU110, GLU2041, ARG2042, ASN2043, GLU2067 **Hydrophobic Interaction:** ILE87, PHE2023, LYS2045, PRO2053	**Hydrogen bond:** LYS137, THR140, ARG164, THR166, GLU167, TYR197, PRO201 **Hydrophobic Interaction:** GLU124, ASP135, LEU136, LYS137, TYR195, PRO201, VAL266	**Hydrogen bond:** LYS39, GLN42, LYS53, GLU61, ASP62, LYS88 **Hydrophobic Interaction:** GLU61, ASP62, THR63, LYS88, LYS88, ILE95	**Hydrogen bond:** SER44, ASP45, ASP95, ARG97, GLN100, GLN302 **Hydrophobic Interaction:** PHE13, PHE16, TYR33, GLU41, TRP96, THR99, GLN302
**Plantaricin BN**	**Hydrogen bond:** PHE161, ASP292, GLY294, LEU295, LEU295 **Hydrophobic Interaction:**	**Hydrogen bond:** THR431, ASN456, LYS458, ASN479, LYS503 **Hydrophobic Interaction:**	**Hydrogen bond:** GLU49, THR52, ASP223, GLN241, GLY259 **Hydrophobic Interaction:**	**Hydrogen bond:** ARG67, THR68, GLU71, ASP150, ASP150, ARG173, ARG173 **Hydrophobic Interaction:**	**Hydrogen bond:** LYS88, ALA112, SER118, GLU2022, ARG2042 **Hydrophobic Interaction:**	**Hydrogen bond:** ASP180, ASN208, SER209, LYS210, TRP214, GLU248	**Hydrogen bond:** ASP62, GLU66 **Hydrophobic Interaction:** ASP62, MET65, GLU66, LYS88	**Hydrogen bond:** GLY40, LYS42, GLN43, GLU117, THR118, ASN261 **Hydrophobic Interaction:**

	PHE161, LEU295<br><br>**Salt Bridge:** LYS179, HIS194	TYR454<br><br>**Salt Bridge:** LYS433	LYS28, PHE239<br><br>**Salt Bridge:** LYS28, ARG50, ARG236, ARG273	LEU171<br><br>**Salt Bridge:** HIS64, HIS174	TYR113, GLU2022<br><br>**Salt Bridge:** LYS88	**Hydrophobic Interaction:** ASP180	**Salt Bridge:** LYS88	LYS42, GLU117<br><br>**Salt Bridge:** ARG229
